# Attribution of Foodborne Illnesses, Hospitalizations, and Deaths to Food Commodities by using Outbreak Data, United States, 1998–2008

**DOI:** 10.3201/eid1903.111866

**Published:** 2013-03

**Authors:** John A. Painter, Robert M. Hoekstra, Tracy Ayers, Robert V. Tauxe, Christopher R. Braden, Frederick J. Angulo, Patricia M. Griffin

**Affiliations:** Author affiliation: Centers for Disease Control and Prevention, Atlanta, GA, USA

**Keywords:** foodborne infections, epidemiology, Salmonella, Shiga toxin–producing Escherichia coli, bacteria, salmonella, E. coli, United States, outbreak data, plans, animals, commodities, commodity groups, food, foodborne illnesses, contamination

## Abstract

Each year, >9 million foodborne illnesses are estimated to be caused by major pathogens acquired in the United States. Preventing these illnesses is challenging because resources are limited and linking individual illnesses to a particular food is rarely possible except during an outbreak. We developed a method of attributing illnesses to food commodities that uses data from outbreaks associated with both simple and complex foods. Using data from outbreak-associated illnesses for 1998–2008, we estimated annual US foodborne illnesses, hospitalizations, and deaths attributable to each of 17 food commodities. We attributed 46% of illnesses to produce and found that more deaths were attributed to poultry than to any other commodity. To the extent that these estimates reflect the commodities causing all foodborne illness, they indicate that efforts are particularly needed to prevent contamination of produce and poultry. Methods to incorporate data from other sources are needed to improve attribution estimates for some commodities and agents.

Despite advances in food safety, foodborne illness remains common in the United States; >9 million persons each year have a foodborne illness caused by a major pathogen (*1*). One challenge in preventing foodborne illness is determining how to prioritize limited food safety resources across a large number of foods (*2*). Furthermore, attributing all illnesses to specific foods is challenging because most agents are transmitted through a variety of foods, and linking an illness to a particular food is rarely possible except during an outbreak.

To help determine priorities for food safety efforts, we organized the large number of foods implicated in outbreaks in the United States into 17 mutually exclusive food commodities. Here, we provide estimates of the number of domestically acquired foodborne illnesses, hospitalizations, and deaths attributable to these commodities.

## Methods

### Data Sources

State and local health departments report foodborne disease outbreaks to the Centers for Disease Control and Prevention (CDC) through the Foodborne Disease Outbreak Surveillance System (*3*). Reports include, when available, number of persons ill, outbreak etiology, description of the implicated food vehicle(s), lists of ingredients, and identification of the contaminated ingredient(s). We reviewed all outbreaks from 1998, the first year with detailed information on ingredients, through 2008 that were reported to the CDC by October 2010. For this analysis, we included all outbreaks with an implicated food vehicle and a single etiologic agent.

Health officials may report whether an etiologic agent was confirmed or suspected on the basis of published criteria (*4*,*5*) and the method of confirmation. Reports may include >1 of 5 reasons for implicating a food vehicle: 1) statistical evidence from an epidemiologic investigation; 2) laboratory evidence identifying the etiologic agent in the implicated food; 3) compelling or other supportive data; 4) previous experience suggesting that the food vehicle is the source; and 5) other data, such as identification of the same etiologic subtype on the farm that supplied the implicated food. We considered an implicated food confirmed when 1 of the first 2 reasons was reported. Other implicated food vehicles were considered suspect. 

To determine whether to analyze outbreaks with suspect foods, we reviewed a convenience sample of 117 outbreak reports for which the reason for implication was not reported. Supporting evidence implicated the food vehicle for 65% of these reports. Some of these outbreaks involved too few persons to conduct an epidemiologic investigation; in most, no food was tested. Outbreaks with suspect vehicles constituted a large proportion of the dataset, but it was not possible to locate and review the documentation for all investigations. However, because a large percentage of documentation reviewed had reasonable evidence to implicate the reported food, we included all outbreaks with suspect foods in the analysis.

During 1998–2008, a total of 13,352 foodborne disease outbreaks, causing 271,974 illnesses, were reported in the United States (Technical Appendix 1 Table 1). Of those outbreaks, 4,887 (37%), causing 128,269 (47%) illnesses, had an implicated food vehicle and a single etiology; 298 of those outbreaks were excluded because information about the vehicle was insufficient to categorize the ingredients. We also did not include the 3% of outbreaks that had multiple etiologies reported. 

To assess possible bias when including outbreaks with a suspected vehicle or etiology in our estimates, we compared the rank order of each of the 17 food commodities in our model based on the total number of associated illnesses with the rank order when including only those illnesses with a confirmed etiology and vehicle. The order of the top 8 commodities associated with the highest number of illnesses changed only slightly (ranks 5 and 6 switched); therefore, we included all outbreaks to maximize the data available for the lower-ranking commodities.

The estimated number of domestically acquired illnesses, hospitalizations, and deaths for each etiology was obtained from published estimates (*1*) or, when not available, by extrapolating from available data. To highlight differences in sources for nontyphoidal *Salmonella* spp. serotypes, we made estimates for those most frequently isolated from humans (i.e., Enteritidis, Heidelberg, Javiana, Newport, Typhimurium) and, separately, for all others. We estimated the number of illnesses, hospitalization, and deaths by multiplying the numbers for nontyphoidal *Salmonella* spp. (*1*) by the proportion of all serotyped human *Salmonella* isolates reported during 1998–2008 (*6*). 

The outbreak dataset included outbreaks with chemical etiologies and those caused by *Anisakis simplex*, for which published illness estimates were not available. For these, the number of illnesses was estimated as the product of the mean annual number of illnesses reported to CDC through outbreak surveillance during 1998–2008 by using the same multipliers for underdiagnosis (×25), underreporting (×30), case-hospitalization rate (×0.006), and case-fatality rate (×0.0004) as for infection with *Clostridium perfringens*, a short-duration illness (*1*).

We attempted to attribute food commodities for an estimated 9,638,301 illnesses, 57,462 hospitalizations, and 1,451 deaths caused by known agents (Technical Appendix 1 Table 2). We did not attribute illnesses to commodities for illnesses caused by astrovirus, *Mycobacterium bovis*, *Toxoplasma gondii*, and *Vibrio vulnificus* because no outbreaks were reported for these pathogens. These pathogens caused an estimated 1.1% of illnesses, 8.1% of hospitalizations, and 25.2% of deaths (a high number of deaths were estimated to be caused by toxoplasmosis [*1*]).

### Food Categorization

**Figure 1:**
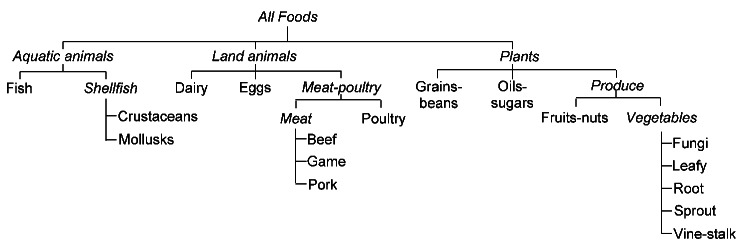
Hierarchy of food commodities. *Italics* indicate commodity groups.

We defined 3 commodities for aquatic animals (fish, crustaceans, and mollusks), 6 for land animals (dairy, eggs, beef, game, pork, and poultry), and 8 for plants (grains-beans; oils-sugars [refined plant foods]; fruits-nuts; fungi; and leafy, root, sprout, and vine-stalk vegetables) (*7*). Foods were categorized into >1 of 17 mutually exclusive commodities according to ingredients listed in outbreak reports, or, when ingredients were not listed, in recipes found on the Internet (*7*). In some analyses, we grouped commodities (Figure 1).

We defined as simple an implicated food vehicle that contained ingredients from 1 commodity, such as apple juice (fruits-nuts commodity). This category included foods such as fruit salad that were composed of several ingredients from the same commodity. We defined as complex an implicated food vehicle that contained ingredients from >1 commodity, such as apple pie (made of ingredients from several commodities: fruits-nuts [apples], grains-beans [flour], oils-sugars [sugar], and dairy [butter]). We excluded water as an ingredient.

### Estimation Method

We calculated for each etiology the proportion of outbreak-associated illnesses transmitted by each commodity. We allocated illnesses from simple food outbreaks of a given etiology to their single implicated commodities. For each complex food outbreak, we partitioned the associated illnesses to the multiple implicated commodities in proportion to the relative numbers of illnesses in all simple food outbreaks that implicated those specific commodities; we then added the results from all outbreaks to obtain commodity illness percentages. We then applied the commodity-specific percentage of ill persons to the total estimated proportion of domestically acquired illnesses, hospitalizations, and deaths for each etiology (*1*). Last, we added the total proportions of commodity-specific illnesses, hospitalizations, and deaths for simple and complex foods for all etiologies. We considered these the most probable estimates for each commodity (Technical Appendix 2).

To provide a range for the most probable estimates, we determined a minimum estimate by attributing illnesses to commodities implicated only in outbreaks where illness was transmitted by simple foods and a maximum estimate by including complex food outbreaks and attributing the outbreak illnesses to each ingredient in the implicated food (Technical Appendix 1 Table 3). Thus, all illnesses in a complex food outbreak with 3 ingredient commodities were included 3 times, once for each commodity. The numbers provided in the Results section are the most probable estimate, unless stated otherwise. Calculations were performed in SAS version 9.3 (SAS Institute, Cary, NC, USA).

## Results

The final dataset consisted of 4,589 outbreaks with an implicated food vehicle and a single etiologic agent (Technical Appendix 3; Technical Appendix 1 Table 1); a total of 120,321 outbreak-associated illnesses were caused by 36 agents (Technical Appendix 1 Table 2). Norovirus caused the most outbreaks (1,419) and outbreak-associated illnesses (41,257), far above the median for all agents (29 outbreaks, 1,208 illnesses). No outbreaks were caused by Mycobacterium bovis, Vibrio vulnificus, astrovirus, or Toxoplasma gondii. The implicated food vehicle was complex for 2,239 (49%) outbreaks (Technical Appendix 1 Table 2); the median number of commodities for complex food vehicles was 4 (range 2–13).

**Figure 2:**
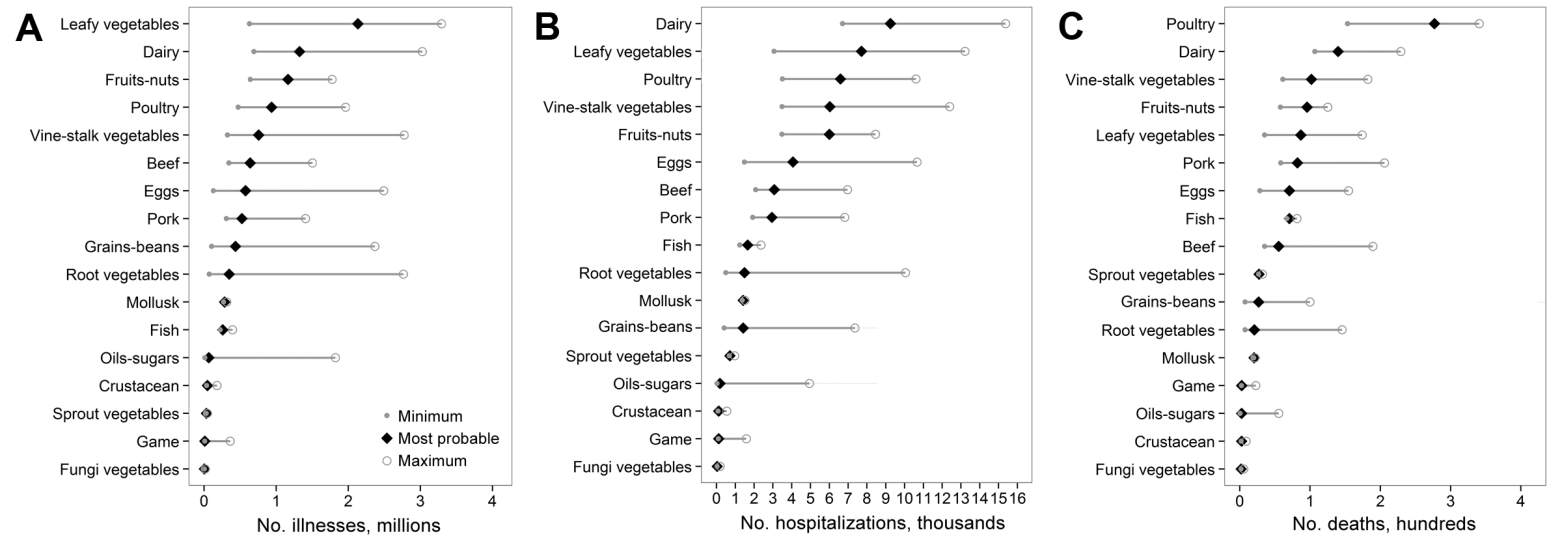
Minimum, most probable, and maximum estimates of the annual number of foodborne illnesses, hospitalizations, and deaths from all etiologies attributed to food commodities, United States, 1998–2008. A) Foodborne illnesses; 102,275 (1.1%) illnesses were not attributed to a commodity and are not shown. B) Foodborne illness–associated hospitalizations; 4,639 (8.1%) hospitalizations were not attributed to a commodity and are not shown. C) Foodborne illness–associated deaths; 366 (25.2%) deaths were not attributed to a commodity and are not shown. Minimum and maximum values represent extreme boundaries for the most probable estimate; they are not the SE of the most probable estimate. For commodities with outbreaks associated with only simple food vehicles, the minimum, maximum, and most probable estimate are the same. For commodities with outbreaks associated with both simple and complex foods, the minimum and maximum estimates reflect the different weighting given to outbreaks associated with complex foods relative to simple. When the most probable estimate for a commodity is close to the minimum estimate, most illnesses from outbreaks associated with complex foods were attributed to another commodity in the food implicated in the outbreak; when the most probable estimate for a commodity is close to the maximum estimate, most illnesses from outbreaks associated with complex foods were attributed to that commodity.

We applied percentages derived from outbreak-associated illnesses for each etiology to the 9.6 million estimated annual illnesses assessed and attributed ≈4.9 million (≈51%) to plant commodities, ≈4.0 million (≈42%) to land animal commodities, and ≈600,000 (≈6%) to aquatic animal commodities (Table 1). Produce commodities (fruits-nuts and the 5 vegetable commodities) accounted for 46% of illnesses; meat-poultry commodities (beef, game, pork, and poultry) accounted for 22%. Among the 17 commodities, more illnesses were associated with leafy vegetables (2.2 million [22%]) than any other commodity. The high estimate for illnesses attributable to leafy vegetables was many times higher than the low estimate (Figure 2, panel A), which indicates that leafy vegetables were frequently found in complex foods. After leafy vegetables, the commodities linked to the most illnesses were dairy (1.3 million [14%]), fruits-nuts (1.1 million [12%]), and poultry (900,000 [10%]). Norovirus comprised 57% of all illnesses.

**Table 1:** Estimates of annual domestically acquired foodborne illnesses attributed to specific food commodities and commodity groups, by pathogen type, United States, 1998–2008*

Commodity or commodity group	No. (%) illnesses				
	All agents	Bacterial	Chemical	Parasitic	Viral
Aquatic animals†	589,310 (6.1)	142,415 (3.9)	153,488 (61.6)	77,795 (33.3)	215,613 (3.9)
Fish	258,314 (2.7)	15,362 (0.4)	148,958 (59.8)	955 (0.4)	93,040 (1.7)
Shellfish†	330,997 (3.4)	127,053 (3.5)	4,531 (1.8)	76,840 (32.9)	122,573 (2.2)
Crustaceans	46,528 (0.5)	32,626 (0.9)	1,247 (0.5)		12,654 (0.2)
Mollusks	284,469 (3.0)	94,427 (2.6)	3,283 (1.3)	76,840 (32.9)	109,919 (2.0)
Land animals†	4,021,839 (41.7)	2,334,000 (64.0)	33,031 (13.3)	156 (0.1)	1,654,651 (30.0)
Dairy	1,330,098 (13.8)	656,951 (18.0)	3,773 (1.5)		669,374 (12.1)
Eggs	574,298 (6.0)	179,421 (4.9)	6,995 (2.8)		387,882 (7.0)
Meat-poultry†	2,117,442 (22.0)	1,497,628 (41.1)	22,263 (8.9)	156 (0.1)	597,394 (10.8)
Meat†	1,174,257 (12.2)	844,006 (23.2)	2,437 (1.0)	156 (0.1)	327,658 (5.9)
Beef	639,640 (6.6)	482,199 (13.2)	661 (0.3)		156,780 (2.8)
Game	9,934 (0.1)	5,111 (0.1)	1,568 (0.6)	156 (0.1)	3,100 (0.1)
Pork	524,684 (5.4)	356,697 (9.8)	209 (0.1)		167,778 (3.0)
Poultry	943,185 (9.8)	653,622 (17.9)	19,826 (8.0)		269,737 (4.9)
Plants†	4,924,877 (51.1)	1,169,202 (32.1)	62,753 (25.2)	69,023 (29.5)	3,623,899 (65.8)
Grains-beans	435,936 (4.5)	183,394 (5.0)	12,995 (5.2)		239,547 (4.3)
Oils-sugars	65,631 (0.7)		2,344 (0.9)		63,287 (1.1)
Produce†	4,423,310 (45.9)	985,807 (27.0)	47,414 (19.0)	69,023 (29.5)	3,321,066 (60.3)
Fruits-nuts	1,123,808 (11.7)	230,636 (6.3)	29,483 (11.8)	60,573 (25.9)	803,116 (14.6)
Vegetables†	3,299,501 (34.2)	755,171 (20.7)	17,931 (7.2)	8,450 (3.6)	2,517,949 (45.7)
Fungi	4,542 (0.0)	686 (0.0)	3,857 (1.5)		
Leafy	2,152,652 (22.3)	188,327 (5.2)	9,113 (3.7)	7,256 (3.1)	1,947,955 (35.4)
Root	349,715 (3.6)	96,910 (2.7)	1,240 (0.5)		251,566 (4.6)
Sprout	32,703 (0.3)	32,703 (0.9)			
Vine-stalk	759,889 (7.9)	436,546 (12.0)	3,721 (1.5)	1,194 (0.5)	318,428 (5.8)
Undetermined	102,275 (1.1)	156 (0.0)		86,686 (37.1)	15,433 (0.3)
Total	9,638,301 (100.0)	3,645,773 (100.0)	249,273 (100.0)	233,660 (100.0)	5,509,596 (100.0)

*Most estimates from (*1*); some were made as described in Methods. Numbers of illnesses are the most probable estimate, as described in Methods. Estimates are rounded; some row and column sums may differ from their totals. Blank cells indicate no data. †Indicates commodity group.

An estimated 26,000 (46%) annual hospitalizations were attributed to land animal commodities, 24,000 (41%) to plant commodities, and 3,000 (6%) to aquatic animal commodities (Table 2). Produce commodities accounted for 38% of hospitalizations and meat-poultry commodities for 22%. Dairy accounted for the most hospitalizations (16%), followed by leafy vegetables (14%), poultry (12%), and vine-stalk vegetables (10%) (Figure 2, panel B). Among the estimated 57,000 hospitalizations, 8% were not attributed to a pathogen, mainly because the dataset did not include data for *Toxoplasma* spp.

**Table 2:** Estimates of annual hospitalizations for domestically acquired foodborne illnesses attributed to specific food commodities and commodity groups, by pathogen type, United States, 1998–2008*

Commodity or commodity group	No. (%) hospitalizations				
	All agents	Bacterial	Chemical	Parasitic	Viral
Aquatic animals†	3,199 (5.6)	1,158 (3.2)	921 (61.6)	231 (4.7)	889 (5.8)
Fish	1,661 (2.9)	210 (0.6)	894 (59.8)	6 (0.1)	551 (3.6)
Shellfish†	1,538 (2.7)	948 (2.6)	27 (1.8)	225 (4.6)	338 (2.2)
Crustaceans	117 (0.2)	75 (0.2)	7 (0.5)		34 (0.2)
Mollusks	1,421 (2.5)	873 (2.4)	20 (1.3)	225 (4.6)	303 (2.0)
Land animals†	26,118 (45.5)	21,471 (60.0)	198 (13.3)	6 (0.1)	4,443 (29.1)
Dairy	9,284 (16.2)	7,464 (20.9)	23 (1.5)		1,798 (11.8)
Eggs	4,062 (7.1)	2,979 (8.3)	42 (2.8)		1,041 (6.8)
Meat-poultry†	12,772 (22.2)	11,029 (30.8)	134 (8.9)	6 (0.1)	1,604 (10.5)
Meat†	6,138 (10.7)	5,238 (14.6)	15 (1.0)	6 (0.1)	880 (5.8)
Beef	3,075 (5.4)	2,650 (7.4)	4 (0.3)		421 (2.8)
Game	117 (0.2)	94 (0.3)	9 (0.6)	6 (0.1)	8 (0.1)
Pork	2,946 (5.1)	2,494 (7.0)	1 (0.1)		450 (2.9)
Poultry	6,634 (11.5)	5,791 (16.2)	119 (8.0)		724 (4.7)
Plants†	23,506 (40.9)	13,043 (36.4)	377 (25.2)	221 (4.5)	9,865 (64.5)
Grains-beans	1,437 (2.5)	695 (1.9)	78 (5.2)		664 (4.3)
Oils-sugars	184 (0.3)		14 (0.9)		170 (1.1)
Produce†	21,885 (38.1)	12,349 (34.5)	284 (19.0)	221 (4.5)	9,031 (59.1)
Fruits-nuts	5,829 (10.1)	3,279 (9.2)	177 (11.8)	213 (4.4)	2,160 (14.1)
Vegetables†	16,057 (27.9)	9,070 (25.3)	108 (7.2)	8 (0.2)	6,871 (45.0)
Fungi	37 (0.1)	14 (0.0)	23 (1.5)		
Leafy	7,769 (13.5)	2,393 (6.7)	55 (3.7)	7 (0.1)	5,314 (34.8)
Root	1,501 (2.6)	793 (2.2)	7 (0.5)		700 (4.6)
Sprout	713 (1.2)	713 (2.0)			
Vine-stalk	6,038 (10.5)	5,157 (14.4)	22 (1.5)	1 (0.0)	857 (5.6)
Undetermined	4,639 (8.1)	124 (0.3)		4,428 (90.6)	87 (0.6)
Total	57,462 (100.0)	35,797 (100.0)	1,496 (100.0)	4,886 (100.0)	15,284 (100.0)

*Most estimates from (*1*); some were made as described in Methods. Numbers of hospitalizations are the most probable estimate, as described in Methods. Estimates are rounded; some row and column sums may differ from their totals. Blank cells indicate no data. †Indicates commodity group.

An estimated 629 (43%) deaths each year were attributed to land animal, 363 (25%) to plant, and 94 (6%) to aquatic commodities (Table 3). Meat-poultry commodities accounted for 29% of deaths and produce 23%. Among the 17 commodities, poultry accounted for the most deaths (19%), followed by dairy (10%), vine-stalk vegetables (7%), fruits-nuts (6%), and leafy vegetables (6%) (Figure 2, panel C). Of the 278 deaths attributed to poultry, most were attributed to *Listeria monocytogenes* (63%) or *Salmonella* spp. (26%). Among the 1,451 estimated deaths, 25% were not attributed to a pathogen, mainly because the dataset did not include data for *Toxoplasma* spp.

**Table 3:** Estimates of annual deaths resulting from domestically acquired foodborne illnesses attributed to specific food commodities and commodity groups, by pathogen type, United States, 1998–2008*

Commodity or commodity group	No. (%) deaths				
	All agents	Bacterial	Chemical	Parasitic	Viral
Aquatic animals†	94 (6.4)	24 (2.8)	61 (61.6)	2 (0.7)	6 (3.7)
Fish	71 (4.9)	8 (1.0)	60 (59.8)	0 (0.1)	2 (1.4)
Shellfish†	23 (1.6)	16 (1.8)	2 (1.8)	2 (0.6)	4 (2.3)
Crustaceans	3 (0.2)	2 (0.2)	0 (0.5)		0 (0.2)
Mollusks	20 (1.4)	14 (1.6)	1 (1.3)	2 (0.6)	3 (2.1)
Land animals†	629 (43.3)	570 (66.2)	13 (13.3)	0	45 (29.0)
Dairy	140 (9.7)	121 (14.0)	2 (1.5)		18 (11.8)
Eggs	71 (4.9)	57 (6.6)	3 (2.8)		11 (6.8)
Meat-poultry†	418 (28.8)	393 (45.5)	9 (8.9)	0	16 (10.4)
Meat†	140 (9.7)	130 (15.1)	1 (1.0)	0	9 (5.7)
Beef	55 (3.8)	51 (5.9)	0 (0.3)		4 (2.7)
Game	3 (0.2)	2 (0.2)	1 (0.6)	0	0 (0.1)
Pork	82 (5.7)	77 (9.0)	0 (0.1)		5 (2.9)
Poultry	278 (19.1)	262 (30.4)	8 (8.0)		7 (4.7)
Plants†	363 (25.0)	229 (26.5)	25 (25.2)	4 (1.2)	105 (67.4)
Grains-beans	27 (1.9)	16 (1.8)	5 (5.2)		6 (4.1)
Oils-sugars	3 (0.2)		1 (0.9)		2 (1.1)
Produce†	333 (22.9)	213 (24.7)	19 (19.0)	4 (1.2)	97 (62.2)
Fruits-nuts	93 (6.4)	55 (6.4)	12 (11.8)	4 (1.2)	22 (14.2)
Vegetables†	240 (16.5)	158 (18.3)	7 (7.2)	0	75 (48.0)
Fungi	2 (0.1)	0	2 (1.5)		
Leafy	88 (6.0)	27 (3.1)	4 (3.7)	0	57 (36.7)
Root	21 (1.4)	12 (1.4)	0 (0.5)		9 (5.6)
Sprout	27 (1.9)	27 (3.2)			
Vine-stalk	102 (7.0)	92 (10.6)	1 (1.5)	0	9 (5.7)
Undetermined	366 (25.2)	39 (4.5)		327 (98.1)	0
Total	1,451 (100.0)	862 (100.0)	100 (100.0)	333 (100.0)	156 (100.0)

*Most estimates from (*1*); some were made as described in Methods. Numbers of deaths are the most probable estimate, as described in Methods. Estimates are rounded; some row and column sums may differ from their totals. Blank cells indicate no data. †Indicates commodity group.

Most bacterial illnesses were attributed to dairy (18%), poultry (18%), and beef (13%) commodities (Table 1). Most chemical illnesses were attributed to fish (60%, most caused by the marine biotoxin ciguatoxin). Most parasitic illnesses were attributed to mollusks (33%) and fruits-nuts (26%); this reflects the fact that 1 simple food outbreak was caused by *Giardia intestinalis* (mollusks) and 1 by *Cryptosporidium* spp. (fruits-nuts). Most viral illnesses were attributed to leafy vegetables (35%), fruits-nuts (15%), and dairy (12%). Of the 20 outbreaks associated with simple foods and caused by norovirus transmitted by dairy, 14 (70%) were transmitted by cheese products.

The plant commodity group accounted for 66% of viral, 32% of bacterial, 25% of chemical, and 30% of parasitic illnesses (Table 1). This group accounted for a greater proportion of illnesses than the land or aquatic animal commodity groups for *Bacillus cereus*; *Clostridium botulinum*; enterotoxigenic *Escherichia coli*; Shiga toxin–producing *Escherichia coli* (STEC) O157; non-O157 STEC; *Salmonella enterica* serotypes Javiana, Newport, and other (e.g., serotypes other than Javiana, Newport, Enteritidis, Heidelberg, Typhimurium, and Typhi); *Shigella* spp.; mycotoxins; other chemicals; *Cryptosporidium* spp.; *Cyclospora cayetansesis*; hepatitis A; norovirus; and sapovirus (Table 4). The land animal group accounted for the highest proportion of illnesses for *Campylobacter* spp., *Clostridium perfringens*, *Listeria* spp., *Salmonella* serotypes Enteritidis and Heidelberg, *Streptococcus* spp. group A, *Yersinia enterocolitica,* and *Trichinella* spp.

**Table 4:** Estimated percentages of foodborne illnesses caused by each pathogen that were attributed to each food commodity, by etiology, United States, 1998–2008*

Etiologic agent (no. outbreaks analyzed)	% Illnesses																			
	Aquatic animals				Land animals							Plants								UND
		Shellfish					Meat-poultry							Produce						
							Meat								Vegetables					
	Fish	Crusta ceans	Mollusk		Dairy	Eggs	Beef	Game	Pork	Poultry		Grains- beans	Oils- sugars	Fruits- nuts	Fungi	Leafy	Root	Sprout	Vine- stalk	
Bacterial (2,468)	0.4	0.9	2.6		18.0	4.9	13.2	0.1	9.8	17.9		5.0		6.3	0.0	5.2	2.7	0.9	12.0	0.0
* Bacillus cereus* (197)	1.0	8.1			0.9		8.6		7.8	16.2		36.8					19.9		0.8	
* Brucella* spp. (4)					100.0															
* Campylobacter* spp. (138)	0.1		6.3		64.8			0.0	1.4	7.6				1.1		8.8	5.8		4.1	
* Clostridium botulinum* (30)	40.3							4.7				9.4			1.8		22.6		21.2	
* Clostridium perfringens* (461)		0.4			0.6	1.2	33.1		7.2	31.3		12.7				0.6	1.3		11.6	
* Escherichia* spp. (206)		5.8			2.3		28.4	0.2	0.4	0.3		0.2		40.5		17.3		0.4	4.2	
ETEC (11)																51.4			48.6	
O157 STEC (186)					7.6		39.4	0.5	1.4	0.8		0.7		21.1		27.3		1.2		
Non-O157 STEC (6)							29.7							62.2		8.1				
Other (3)		100.0																		
* Listeria monocytogenes* (21)					15.9		2.2		7.5	68.4								6.0		
* Mycobacterium bovis* (0)																				100.0
* Salmonella enterica* (877)	0.7	0.2	0.2		7.2	14.8	7.3	0.4	6.2	19.0		2.9		13.0	0.1	2.9	1.2	3.1	20.7	
ser. Enteritidis (284)	0.3	0.6			1.2	60.3	2.0		1.0	17.4				3.3		1.3		1.3	11.3	
ser. Heidelberg (66)						37.6	9.6			24.3				28.6						
ser. Javiana (17)									10.1	1.4				48.4		6.7	5.4		28.1	
ser. Newport (58)					9.1		12.2		5.8	10.1				11.5		7.4	5.8		38.2	
ser. Typhimurium (106)	1.8				23.3	2.8	7.1	0.8	8.2	34.0					0.3	5.3	0.7	4.1	11.6	
ser. Typhi (2)	0.8	0.1			3.5	5.7	8.8	0.5	7.5	15.9		6.3		17.8		1.5	0.8	4.5	26.2	
Other (344)			100.0																.	
* Shigella* spp. (62)	1.4		0.2		2.9	3.5	3.2		0.1	2.2		2.1		2.6		32.6			49.4	
* Staphylococcus aureus* (384)	0.3	0.7	0.4		7.5	4.5	7.7	0.2	44.7	27.0		1.8					4.4		1.0	
* Streptococcus* spp. group A (1)										100.0										
* Vibrio* spp. (80)	7.4	16.7	72.9							2.8										0.2
* V. cholerae*, toxigenic (3)		50.0	50.0																	
* V. parahaemolyticus* (68)		25.1	70.6							4.2										
* V. vulnificus* (0)																				100.0
Other (9)	22.2		77.8																	
* Yersinia enterocolitica* (7)	.				.	.	.	.	100.0											
Chemical (632)	59.8	0.5	1.3		1.5	2.8	0.3	0.6	0.1	8.0		5.2	0.9	11.8	1.5	3.7	0.5		1.5	
Marine biotoxins (527)	97.3	0.5	2.2																	
Mycotoxins (16)														58.6	41.4					
Other chemicals (89)	5.4	0.5	.		4.1	7.6	0.7	1.7	0.2	21.4		14.0	2.5	26.3	0.3	9.8	1.3		4.0	
Parasitic (33)	0.4		32.9					0.1						25.9		3.1			0.5	37.1
* Anisakis simplex* (1)	100.0																			
* Cryptosporidium* spp. (3)														100.0		.				
* Cyclospora cayetanensis* (16)														25.9		63.6			10.5	
* Giardia intestinalis* (4)			100.0																	
* Toxoplasma gondii* (0)																				100.0
* Trichinella* spp. (9)								100.0												
Viral	1.7	0.2	2.0		12.1	7.0	2.8	0.1	3.0	4.9		4.3	1.1	14.6		35.4	4.6	.	5.8	0.3
Astrovirus (0)																				100.0
Hepatitis A virus (29)		0.6	3.1		1.0									4.2		63.2	26.0		2.0	
Norovirus (1,418)	1.4	0.2	2.0		12.3	7.1	2.9	0.1	3.1	4.9		4.3	1.2	14.7		35.5	4.6		5.8	
Rotavirus (5)	98.3		1.7																	
Sapovirus (2)												46.2				53.8				
Total (4,587)	2.7	0.5	3.0		13.8	6.0	6.6	0.1	5.4	9.8		4.5	0.7	11.7	0.0	22.3	3.6	0.3	7.9	1.1

*See online Technical Appendix 1 Table 2 (wwwnc.cdc.gov/EID/article/19/3/11-1866-Techapp1.pdf) for breakdown of outbreaks by those involving simple food or complex food, number of outbreak-associated illnesses, and estimated annual number of illnesses used to determine the percentages. A value of 0.0 indicates percentage below 0.1%; blank cells indicate no illnesses in that category. UND, undetermined; ser., serotype; STEC, Shiga toxin–producing *Escherichia coli*; ETEC, enterotoxigenic* E. coli*; other, diarrheagenic *E. coli* other than STEC or ETEC.

## Discussion

We developed a method to attribute domestically acquired foodborne illnesses, hospitalizations, and deaths in the United States to specific commodities by using outbreak data. We found most illnesses were attributed to plant commodities and most deaths to land animal commodities. We attributed 46% of illnesses to produce; the large number of norovirus illnesses was a major driver of this result. More deaths were attributed to poultry than to any other commodity. To the extent that these outbreak-based estimates reflect the commodities associated with all foodborne illness, they indicate that efforts are particularly needed to prevent contamination of produce and poultry.

More illnesses were attributed to leafy vegetables (22%) than to any other commodity; illnesses associated with leafy vegetables were the second most frequent cause of hospitalizations (14%) and the fifth most frequent cause of death (6%). Previous studies have shown that produce-containing foods were the food source for approximately half of norovirus outbreaks with an identified simple food vehicle during 2001–2008 (*8*) and the second most frequent food source for *E. coli* O157 outbreaks during 1982–2002 (*9*). Outbreaks of *E. coli* O157 infections transmitted by spinach (*10*) and lettuce (*11*) and *Salmonella* spp. infections transmitted by tomatoes (*12*,*13*), juice (*14*,*15*), mangoes (*16*), sprouts (*17*,*18*), and peppers (*19*,*20*) underline concerns about contamination of produce consumed raw.

More deaths were attributed to poultry (19%) than to any other commodity, and most poultry-associated deaths were caused by *Listeria* or *Salmonella* spp. From 1998 through 2002, three large listeriosis outbreaks were linked to turkey delicatessen meat contaminated in the processing plant after cooking (*21*–*23*). A risk-ranking model for listeriosis among ready-to-eat foods identified delicatessen meat as the highest risk food (*24*).

The dairy commodity was the second most frequent food source for infections causing illnesses (14%) and deaths (10%). Foods in this commodity are typically consumed after pasteurization, which eliminates pathogens, but improper pasteurization and incidents of contamination after pasteurization occur (*25*). In our dataset, norovirus outbreaks associated with cheese illustrate the role of contamination of dairy products after pasteurization by food handlers. Because of the large volume of dairy products consumed, even infrequent contamination of commercially distributed products can result in many illnesses (*26*). The prominence of dairy in our model reflects a relatively high number of reported outbreaks associated with raw milk compared with the quantity of raw milk consumed (*27*) and issues related to *Campylobacter* spp. infection (discussed below); these factors likely resulted in an overestimation of illnesses attributed to dairy. Models that partition raw versus pasteurized milk and that incorporate other data sources for *Campylobacter* spp. infection could improve estimates of illnesses related to dairy.

Our method of attributing illnesses incorporated data from outbreaks associated with complex foods and attributed most of the estimated number of illnesses caused by known pathogens to specific food sources. Other methods for attributing illnesses to food sources may be applied to various stages of the food distribution chain and therefore may yield different but complementary estimates (*2*). A method for *Salmonella* spp. attribution used in Denmark compared isolates from food animal reservoirs with human isolates to attribute infections to the reservoirs, the live animals (*28*). A similar method in a US study attributed *Salmonella* spp.–associated foodborne illnesses to the point of processing (*29*). Risk assessment models have focused primarily on the point of processing; case studies of sporadic illness, expert elicitation, and analysis of outbreak data represent attribution at the point of consumption. Outbreak investigations have been reported for most foodborne etiologies and food commodities and provide the most comprehensive data for attribution.

We made several assumptions. We assumed that using the number of outbreak-associated illnesses rather than number of outbreaks would enable better assignment of illnesses to commodities. Our choice had the potential to bias the results toward large outbreaks. However, large outbreaks often represent system failures that have resulted in smaller, undetected outbreaks; investigation may determine the source for illnesses that otherwise might have been considered sporadic. Small outbreaks may better represent sources of sporadic illnesses, but because many small outbreaks are not detected or investigated, their sources would not be well represented by any method. Similar studies have used outbreak counts (*30*,*31*); either choice (number of outbreak-associated illnesses or number of outbreaks) results in biases (*32*). Because of other methodological differences, direct comparison of the results for these studies is difficult. To assess the effect of outbreak size on our estimates, we adjusted our model to give no weight to outbreak size (Technical Appendix 1 Tables 4, 5); the rank order of commodities by number of attributed illnesses changed by no more than 1 for most commodities. The largest outbreak in our study was 1,644 *Campylobacter* spp.–associated illnesses resulting from the consumption of pasteurized milk; even so, counting outbreaks instead of illnesses resulted in a relatively small (2.6%) reduction in the percentage of illnesses attributed to dairy.

We further assumed outbreak illnesses represented all illnesses and weighted the results for each agent by number of all foodborne illnesses attributed to each agent (*1*). Unweighted outbreak data may be biased toward seafood outbreaks caused by marine biotoxins (e.g., scombroid) that are frequently reported but cause relatively few illnesses. For some agents, foods implicated in outbreaks might not well represent foods responsible for sporadic illnesses. For example, outbreak data underrepresent poultry (8%) and overrepresent dairy (67%) as sources of *Campylobacter* spp. infection; studies of sporadic infections implicate consumption of poultry but not dairy as a major risk factor (*33*). *Campylobacter* spp. are estimated to be the third most common bacterial cause of foodborne illness, but relatively few outbreaks are detected (*1*). For pathogens for which outbreaks are uncommon or do not reflect major modes of transmission, methods that incorporate data from nonoutbreak sources are needed.

We also assumed that, for a given agent, when an outbreak was associated with a complex food, the likelihood that any commodity was the source was proportional to the frequency of illnesses for outbreaks associated with simple foods associated with that commodity. However, when the number of outbreaks associated with simple foods for an etiology is small compared with the number associated with complex foods, the result may be biased toward commodities for which simple foods were vehicles for outbreaks. Other attribution estimates that used outbreak surveillance data have excluded complex foods or have not partitioned them into component commodities (*9*,*34*). Were complex food outbreaks excluded, the result for each commodity would be the same as our minimum estimate. However, inclusion of outbreaks associated with complex foods provides important information. For example, in a review of egg-associated *S. enterica* serotype Enteritidis outbreaks (*35*), eggs were implicated as simple food vehicles in 20% of the outbreaks, but complex foods containing eggs were implicated in an additional 57% of the outbreaks.

A limitation of our study is the absence of outbreaks caused by some agents. None caused by *Toxoplasma* spp. or *Vibrio vulnificus* were reported. The attributable risk for *Toxoplasma* infection is highest for meat (49%) and mollusks (16%) (*36*); most foodborne *V. vulnificus* infections are linked to oysters (*37*). The effect of this absence of data for agents that are uncommon but often cause fatal illnesses is reflected mostly in the number of deaths in our study, 25% of which were not attributed. Attributing an additional 49% of *Toxoplasma* spp.–associated deaths to meats would make meats a more frequent source of foodborne illness–associated deaths than poultry. Attributing all foodborne deaths caused by *V. vulnificus* and 16% of those caused by *Toxoplasma* spp. to mollusks would move this commodity from the thirteenth to the fourth most frequent source of foodborne illness–associated deaths.

Other limitations of our study included the choice not to use the credible interval for the estimated number of illnesses, hospitalization, and deaths (*1*); the lack of published estimates for the number of illnesses caused by chemical etiologies; and the fact that the quality of outbreak data is dependent on the quality and quantity of investigations reported. We maximized the amount of data we compiled by including outbreaks with suspect etiologies or vehicles and developing a method to incorporate data from outbreaks attributed to both simple and complex foods; even so, our study yielded a paucity of data for some agents. Among the agents associated with <10 outbreaks in the dataset, only 1 (non-O157 STEC) is estimated to cause >1% of foodborne illnesses caused by known agents (*1*). Our estimates should be considered an approximation, to be refined by further research and analyses. To improve the quality and accuracy of outbreak attribution, models can be developed that include other types of data (e.g., studies of sporadic cases, isolates from foods and animals, agent subtypes). Measurements that indicate the substantial uncertainty of many of the estimates are particularly critical for agents causing few outbreaks and those for which the major sources for outbreaks are dissimilar to those for sporadic cases. Ultimately, the best data sources and methods for estimating the number of illnesses, hospitalizations, and deaths attributable to each food commodity may vary by etiologic agent, commodity, point of food chain analyzed, and other factors.

For consistency and to obtain sufficient data, we chose to use all years of data for all pathogens, but a shorter, more recent period is desirable when major implicated commodities have changed. For example, outbreaks of *Listeria* spp. infection caused by contamination of ready-to-eat meats markedly decreased after 2002 (*38*). However, using data from only the few listeriosis outbreaks that occurred after 2002 would result in a few commodities having a large effect on results. Developing methods to examine trends should be a high priority. When combined with updated estimates of the number of illnesses, attribution analyses performed at appropriate intervals could help determine the results of prevention efforts. Longer intervals would increase data for agents with few outbreaks, but if the frequency of illness attributed to a commodity changes substantially, results might not reflect the current situation.

In summary, our outbreak-based method attributed most foodborne illnesses to food commodities that constitute a major portion of the US diet. When food commodities are consumed frequently, even those with a low risk for pathogen transmission per serving may result in a high number of illnesses. The attribution of foodborne-associated illnesses and deaths to specific commodities is useful for prioritizing public health activities; however, additional data on the specific food consumed is needed to assess per-serving risk. The risk for foodborne illness is just one part of the risk–benefit equation for foods; other factors, such as the health benefits of consuming a diet high in fruits and vegetables, must also be considered (*39*).

Technical Appendix 1 Analyses of foodborne disease outbreaks, United States, 1998–2008: number of simple or complex implicated food vehicles; estimated annual number of illnesses, hospitalizations, and deaths; minimum and maximum percentages of annual foodborne illnesses caused by each agent that were attributed to each food commodity; number of foodborne disease outbreaks that were attributed to each food commodity; and comparison of rank order of illnesses, hospitalizations, and deaths attributed to food commodities.

Technical Appendix 2 Additional methods related to attributing human illnesses to food commodities.

Technical Appendix 3 Foodborne disease outbreaks attributed to each food commodity, by etiologic agent and number of commodities, United States, 1998–2008.

## References

[1] Scallan E, Hoekstra RM, Angulo FJ, Tauxe RV, Widdowson MA, Roy SL, Foodborne illness acquired in the United States—major pathogens. Emerg Infect Dis. 2011;17:7–15. 10.3201/eid1701.P11101 21192848 PMC3375761

[2] Batz MB, Doyle MP, Morris G Jr, Painter J, Singh R, Tauxe RV, Attributing illness to food. Emerg Infect Dis. 2005;11:993–9. 10.3201/eid1107.040634 16022770 PMC3371809

[3] Centers for Disease Control and Prevention. Outbreak surveillance data. 2010 [cited 2012 Jun 22]. http://www.cdc.gov/outbreaknet/surveillance_data.html

[4] Lynch M, Painter J, Woodruff R, Braden C. Surveillance for foodborne-disease outbreaks—United States, 1998–2002. MMWR Surveill Summ. 2006;55:1–42. 17093388

[5] Olsen SJ, MacKinnon LC, Goulding JS, Bean NH, Slutsker L. Surveillance for foodborne-disease outbreaks—United States, 1993–1997. MMWR CDC Surveill Summ. 2000;49:1–62. 10789699

[6] Centers for Disease Control and Prevention. *Salmonella* surveillance: annual summary. 2008 [cited 2012 Jun 22]. http://www.cdc.gov/ncidod/dbmd/phlisdata/salmonella.htm

[7] Painter JA, Ayers T, Woodruff R, Blanton E, Perez N, Hoekstra RM, Recipes for foodborne outbreaks: a scheme for categorizing and grouping implicated foods. Foodborne Pathog Dis. 2009;6:1259–64. 10.1089/fpd.2009.0350 19968563

[8] Hall AJ, Eisenbart VG, Etingue AL, Gould LH, Lopman BA, Parashar UD. Epidemiology of foodborne norovirus outbreaks, United States, 2001–2008. Emerg Infect Dis. 2012;18:1566–73. 10.3201/eid1810.120833 23017158 PMC3471645

[9] Rangel JM, Sparling PH, Crowe C, Griffin PM, Swerdlow DL. Epidemiology of *Escherichia coli* O157:H7 outbreaks, United States, 1982–2002. Emerg Infect Dis. 2005;11:603–9. 10.3201/eid1104.040739 15829201 PMC3320345

[10] Centers for Disease Control and Prevention. Ongoing multistate outbreak of *Escherichia coli* serotype O157:H7 infections associated with consumption of fresh spinach—United States, September 2006. MMWR Morb Mortal Wkly Rep. 2006;55:1045–6. 17008868

[11] Hilborn ED, Mermin JH, Mshar PA, Hadler JL, Voetsch A, Wojtkunski C, A multistate outbreak of *Escherichia coli* O157:H7 infections associated with consumption of mesclun lettuce. Arch Intern Med. 1999;159:1758–64. 10.1001/archinte.159.15.1758 10448779

[12] Centers for Disease Control and Prevention. Multistate outbreaks of *Salmonella* infections associated with raw tomatoes eaten in restaurants—United States, 2005–2006. MMWR Morb Mortal Wkly Rep. 2007;56:909–11. 17805221

[13] Greene SK, Daly ER, Talbot EA, Demma LJ, Holzbauer S, Patel NJ, Recurrent multistate outbreak of *Salmonella* Newport associated with tomatoes from contaminated fields, 2005. Epidemiol Infect. 2008;136:157–65. 10.1017/S095026880700859X 17475091 PMC2870807

[14] Jain S, Bidol SA, Austin JL, Berl E, Elson F, Lemaile-Williams M, Multistate outbreak of *Salmonella* Typhimurium and Saintpaul infections associated with unpasteurized orange juice—United States, 2005. Clin Infect Dis. 2009;48:1065–71. 10.1086/597397 19281328

[15] Vojdani JD, Beuchat LR, Tauxe RV. Juice-associated outbreaks of human illness in the United States, 1995 through 2005. J Food Prot. 2008;71:356–64. 18326187 10.4315/0362-028x-71.2.356

[16] Sivapalasingam S, Barrett E, Kimura A, Van Duyne S, De Witt W, Ying M, A multistate outbreak of *Salmonella enterica* Serotype Newport infection linked to mango consumption: impact of water-dip disinfestation technology. Clin Infect Dis. 2003;37:1585–90. 10.1086/379710 14689335

[17] Brooks JT, Rowe SY, Shillam P, Heltzel DM, Hunter SB, Slutsker L, *Salmonella* Typhimurium infections transmitted by chlorine-pretreated clover sprout seeds. Am J Epidemiol. 2001;154:1020–8. 10.1093/aje/154.11.1020 11724718

[18] Winthrop KL, Palumbo MS, Farrar JA, Mohle-Boetani JC, Abbott S, Beatty ME, Alfalfa sprouts and *Salmonella* Kottbus infection: a multistate outbreak following inadequate seed disinfection with heat and chlorine. J Food Prot. 2003;66:13–7. 12540175 10.4315/0362-028x-66.1.13

[19] Barton Behravesh C, Mody RK, Jungk J, Gaul L, Redd JT, Chen S, 2008 outbreak of *Salmonella* Saintpaul infections associated with raw produce. N Engl J Med. 2011;364:918–27. 10.1056/NEJMoa1005741 21345092

[20] Mody RK, Greene SA, Gaul L, Sever A, Pichette S, Zambrana I, National outbreak of *Salmonella* serotype Saintpaul infections: importance of Texas restaurant investigations in implicating jalapeno peppers. PLoS ONE. 2011;6:e16579. 10.1371/journal.pone.0016579 21373185 PMC3044132

[21] Gottlieb SL, Newbern EC, Griffin PM, Graves LM, Hoekstra RM, Baker NL, Multistate outbreak of listeriosis linked to turkey deli meat and subsequent changes in US regulatory policy. Clin Infect Dis. 2006;42:29–36. 10.1086/498113 16323088

[22] Mead PS, Dunne F, Graves L, Wiedmann M, Patrick M, Hunter S, Nationwide outbreak of listeriosis due to contaminated meat. Epidemiol Infect. 2006;134:744–51. 10.1017/S0950268805005376 16318652 PMC2870438

[23] Olsen SJ, Patrick M, Hunter SB, Reddy V, Kornstein L, MacKenzie WR, Multistate outbreak of *Listeria monocytogenes* infection linked to delicatessen turkey meat. Clin Infect Dis. 2005;40:962–7. 10.1086/428575 15824987

[24] Food and Drug Administration. Quantitative assessment of relative risk to public health from foodborne *Listeria monocytogenes* among selected categories of ready-to-eat foods. 2003 Sep [cited 2012 Dec 20]. http://www.fda.gov/downloads/food/scienceresearch/researchareas/riskassessmentsafetyassessment/ucm197330.pdf

[25] Olsen SJ, Ying M, Davis MF, Deasy M, Holland B, Iampietro L, Multidrug-resistant *Salmonella* Typhimurium infection from milk contaminated after pasteurization. Emerg Infect Dis. 2004;10:932–5. 10.3201/eid1005.030484 15200835 PMC3323239

[26] Ryan CA, Nickels MK, Hargrett-Bean NT, Potter ME, Endo T, Mayer L, Massive outbreak of antimicrobial-resistant salmonellosis traced to pasteurized milk. JAMA. 1987;258:3269–74. 10.1001/jama.1987.03400220069039 3316720

[27] Langer AJ, Ayers T, Grass J, Lynch M, Angulo FJ, Mahon BE. Nonpasteurized dairy products, disease outbreaks, and state laws-United States, 1993–2006. Emerg Infect Dis. 2012;18:385–91. 10.3201/eid1803.111370 22377202 PMC3309640

[28] Hald T, Vose D, Wegener HC, Koupeev T. A Bayesian approach to quantify the contribution of animal-food sources to human salmonellosis. Risk Anal. 2004;24:255–69. 10.1111/j.0272-4332.2004.00427.x 15028016

[29] Guo C, Hoekstra RM, Schroeder CM, Pires SM, Ong KL, Hartnett E, Application of Bayesian techniques to model the burden of human salmonellosis attributable to U.S. Food commodities at the point of processing: adaptation of a Danish model. Foodborne Pathog Dis. 2011;8:509–16. 10.1089/fpd.2010.0714 21235394 PMC3123837

[30] Batz MB, Hoffmann S, Morris JG. Ranking the disease burden of 14 pathogens in food sources in the United States using attribution data from outbreak investigations and expert elicitation. J Food Prot. 2012;75:1278–91. 10.4315/0362-028X.JFP-11-418 22980012

[31] Dewall CS, Hicks G, Barlow K, Alderton L, Vegosen L. Foods associated with foodborne illness outbreaks from 1990 through 2003. Food Prot Trends. 2006;26:466–73.

[32] Pires SM, Vieira AR, Perez E, Lo Fo Wong D, Hald T. Attributing human foodborne illness to food sources and water in Latin America and the Caribbean using data from outbreak investigations. Int J Food Microbiol. 2012;152:129–38. 10.1016/j.ijfoodmicro.2011.04.018 21570732

[33] Friedman CR, Hoekstra RM, Samuel M, Marcus R, Bender J, Shiferaw B, Risk factors for sporadic *Campylobacter* infection in the United States: a case-control study in FoodNet sites. Clin Infect Dis. 2004;38(Suppl 3):S285–96. 10.1086/381598 15095201

[34] Adak GK, Meakins SM, Yip H, Lopman BA, O’Brien SJ. Disease risks from foods, England and Wales, 1996–2000. Emerg Infect Dis. 2005;11:365–72. 15757549 10.3201/eid1103.040191 PMC3298246

[35] St Louis ME, Morse DL, Potter ME, DeMelfi TM, Guzewich JJ, Tauxe RV, The emergence of grade A eggs as a major source of *Salmonella* Enteritidis infections. New implications for the control of salmonellosis. JAMA. 1988;259:2103–7. 10.1001/jama.259.14.2103 3279240

[36] Jones JL, Dargelas V, Roberts J, Press C, Remington JS, Montoya JG. Risk factors for *Toxoplasma gondii* infection in the United States. Clin Infect Dis. 2009;49:878–84. 10.1086/605433 19663709

[37] Altekruse SF, Bishop RD, Baldy LM, Thompson SG, Wilson SA, Ray BJ, *Vibrio* gastroenteritis in the US Gulf of Mexico region: the role of raw oysters. Epidemiol Infect. 2000;124:489–95. 10.1017/S0950268899003714 10982073 PMC2810935

[38] Cartwright EJ, Jackson KA, Johnson SD, Graves LM, Silk BJ, Mahon BE. Listeriosis outbreaks and associated food vehicles, United States, 1998–2008. Emerg Infect Dis. 2013;19:1–9. 10.3201/eid1901.120393 23260661 PMC3557980

[39] US Department of Agriculture; US Department of Health and Human Services. Dietary guidelines for Americans, 2010. 7th ed. 2010 Dec [cited 2012 Dec 20]. http://www.health.gov/dietaryguidelines/dga2010/dietaryguidelines2010.pdf

