# Supplementary material for: Attribution of Foodborne Illnesses, Hospitalizations, and Deaths to Food Commodities by using Outbreak Data, United States, 1998–2008
Source: Emerg Infect Dis. 2013 Mar;19(3):407–15. doi: 10.3201/eid1903.111866 (PMC3647642; doi:10.3201/eid1903.111866)
Supplement: Technical Appendix 1 — Analyses of foodborne disease outbreaks, United States, 1998–2008: number of simple or complex implicated food vehicles; estimated annual number of illnesses, hospitalizations, and deaths; minimum and maximum percentages of annual foodborne illnesses caused by each agent that were attributed to each food commodity; number of foodborne disease outbreaks that were attributed to each food commodity; and comparison of rank order of illnesses, hospitalizations, and deaths attributed to food commodities. [file 11-1866-Techapp-s1.pdf]

Article DOI: http://dx.doi.org/10.3201/eid1903.111866

Attribution of Foodborne Illnesses, Hospitalizations, and Deaths to Food Commodities Using Outbreak Data, United States, 1998–2008

Technical Appendix 1

Technical Appendix 1 Table 1. Foodborne disease outbreaks reported to CDC, 1998–2008. Shaded area indicates the categories of outbreaks (N=4,887) included in this analysis. Of these, 298 outbreaks were not included in analysis because information about the vehicle was insufficient to categorize the ingredient commodities.

	Food vehicle															
	Confirmed				Suspected				Unidentified				TOTAL			
	Outbreaks		Illnesses		Outbreaks		Illnesses		Outbreaks		Illnesses		Outbreaks		Illnesses	
Etiologic Agent	No.	(%)	No.	(%)	No.	(%)	No.	(%)	No.	(%)	No.	(%)	No.	(%)	No.	(%)
Single, confirmed	1,811	(57)	73,103	(68)	1,285	(28)	27,145	(49)	1,908	(34)	54,869	(50)	5,004	(37)	155,117	(57)
Single, suspected	560	(18)	14,914	(14)	1,231	(27)	13,107	(24)	962	(17)	16,247	(15)	2,753	(21)	44,268	(16)
Multiple	120	(4)	4,998	(5)	121	(3)	1,805	(3)	126	(2)	1,490	(1)	367	(3)	8,293	(3)
Unidentified	698	(22)	14,676	(14)	1,884	(42)	13,251	(24)	2,646	(47)	36,369	(33)	5,228	(39)	64,296	(24)
TOTAL	3,189	(100)	107,691	(100)	4,521	(100)	55,308	(100)	5,642	(100)	108,975	(100)	13,352	(100)	271,974	(100)

Technical Appendix 1 Table 2. The number of foodborne disease outbreaks, with simple or complex implicated food vehicles, 1998–2008, and the estimated annual number of illnesses, hospitalizations, and deaths, by etiology

	Reported Outbreaks			Reported outbreak-associated Illnesses			Estimated Annual Numbers*		
Etiologic Agent	Total	Simple food	Complex food	Total	Simple food	Complex food	Illnesses	Hosp. †	Deaths
Bacterial	2,469	1,301	1,168	72,890	40,107	32,783	3,645,773	35,797	862
Bacillus cereus	197	74	123	1,647	699	948	63,400	20	0
Brucella spp.	4	4	.	14	14	.	839	55	1
Campylobacter spp.	138	105	33	4,395	3,911	484	845,024	8,463	76
Clostridium botulinum	30	20	10	111	62	49	55	42	9
Clostridium perfringens	461	234	227	18,710	9,470	9,240	965,958	438	26
Escherichia	206	138	68	6,778	4,382	2,396	205,781	2,429	21
E. coli, ETEC†	11	3	8	1,878	466	1,412	17,894	12	0
E. coli, O157 STEC†	186	128	58	4,844	3,867	977	63,153	2,138	20
E. coli, non-O157 STEC†	6	6	.	37	37	.	112,752	271	1
E. coli, other†	3	1	2	19	12	7	11,982	8	0
Listeria monoctyogenes	21	15	6	336	217	119	1,591	1,455	255
Mycobacterium bovis	.	.	.	.	.	.	60	31	3
Salmonella enterica	877	482	395	29,685	16,000	13,685	1,029,382	19,533	378
Ser. Enteritidis	284	149	135	8,627	4,629	3,998	168,041	3,162	62
Ser. Heidelberg	66	23	43	3,151	456	2,695	49,478	931	18
Ser. Javiana	17	11	6	1,279	916	363	40,337	759	15
Ser. Newport	58	40	18	2,280	1,903	377	95,119	1,790	35
Ser. Typhimurium	106	59	47	4,113	1,767	2,346	202,497	3,810	74
S. spp., other non-typhoidal	344	199	145	10,213	6,323	3,890	472,089	8,883	174
Ser. Typhi	2	1	1	22	6	16	1,821	197	0
Shigella spp.	63	25	38	3,875	1,808	2,067	131,254	1,456	10
Staphylococcus aureus	384	135	249	6,032	2,356	3,676	241,148	1,063	6
Streptococcus spp. group A	1	1	.	4	4	.	11,217	1	0
Vibrio	80	62	18	1,238	1,128	110	52,408	278	48
V. cholerae, toxigenic	3	3	.	12	12	.	84	2	0
V. parahaemolyticus	68	51	17	1,208	1,100	108	34,664	100	4
V. vulnificus	.	.	.	.	.	.	96	93	36
V. spp., other	9	8	1	18	16	2	17,564	83	8
Yersinia enterocolitica	7	6	1	65	56	9	97,656	533	29
Chemical	632	573	59	3,235	2,746	489	249,273	1,496	100
Marine biotoxins	527	513	14	2,170	2,129	41	147,955	888	59
Mycotoxins	16	15	1	128	121	7	8,727	52	3
Other chemicals	89	45	44	937	496	441	92,591	556	37
Parasitic	33	22	11	1,449	1,156	293	233,660	4,886	333
Anisakis simplex	1	1	.	14	14	.	955	6	0
Cryptosporidium spp.	3	1	2	157	144	13	57,616	210	4
Cyclospora cayetanensis	16	12	4	1,164	965	199	11,407	11	0
Giardia intestinalis	4	1	3	74	3	71	76,840	225	2
Toxoplasma gondii	.	.	.	.	.	.	86,686	4,428	327
Trichinella spp.	9	7	2	40	30	10	156	6	0
Viral	1,455	448	1,007	42,747	13,113	29,634	5,509,596	15,284	156
Astrovirus	.	.	.	.	.	.	15,433	87	0
Hepatitis A virus	29	13	16	1,303	1,145	158	1,566	99	7

Etiologic Agent	Reported Outbreaks: Total	Simple food	Complex food	Reported outbreak-associated Illnesses: Total	Simple food	Complex food	Estimated Annual Numbers*: Illnesses	Hosp.†	Deaths
Norovirus	1,419	431	988	41,257	11,922	29,335	5,461,731	14,663	149
Rotavirus	5	2	3	148	7	141	15,433	348	0
Sapovirus	2	2	.	39	39	.	15,433	87	0
Total	4,589	2,344	2,245	120,321	57,122	63,199	9,638,301	57,462	1,451

*The values not previously published[1] were estimated as described in methods.
†Hosp.=Hospitalizations, STEC=Shiga toxin-producing *Escherichia coli,* ETEC=Enterotoxigenic *Escherichia coli,* other= diarrheagenic other than STEC and ETEC

Technical Appendix 1 Table 3. Minimum and maximum percentages of annual U.S. foodborne illnesses caused by each agent that were attributed to each food commodity, by etiologic agent, using outbreak data from 1998 through 2008

	Fish		Crust.		Mollusks		Dairy		Eggs		Beef		Game		Pork		Poultry		Grains-Beans		Oils-Sugars		Fruits-Nuts		Fungi		Leafy		Root		Sprout		Vine-Stalk	
Etiologic Agent	min	max	min	max	min	max	min	max	min	max	min	max	min	max	min	max	min	max	min	max	min	max	min	max	min	max	min	max	min	max	min	max	min	max
Bacterial	0.0	0.2	0.1	0.3	0.4	0.4	2.3	4.1	0.3	1.6	1.1	2.6	0.0	0.2	1.0	2.3	1.5	3.4	0.2	1.9	.	.	0.7	1.3	0.0	0.0	0.5	1.8	0.1	2.2	0.1	0.2	1.0	2.7
Bacillus cereus	0.5	5.6	7.2	10.0	.	.	0.2	12.8	.	.	5.4	13.9	.	.	5.7	13.4	7.9	29.1	10.7	53.0	.	.	0.4	5.0	.	.	.	.	4.2	42.9	.	.	0.2	14.5
Brucella spp.	.	.	.	.	.	.	100	100	.	.	.	.	.	.	.	.	.	.	.	.	.	.	.	.	.	.	.	.	.	.	.	.	.	.
Campylobacter spp.	0.1	0.2	.	.	6.2	6.3	61.8	65.2	.	.	.	.	0.0	0.6	0.6	6.3	5.3	10.3	.	.	.	.	0.7	5.4	.	.	8.4	10.4	3.1	9.1	.	.	2.8	6.2
Clostridium botulinum	35.1	40.5	.	.	.	.	.	.	.	.	.	.	3.6	7.2	.	.	.	.	1.8	12.6	.	.	.	.	1.8	1.8	.	.	5.4	35.1	.	.	8.1	29.7
Clostridium perfringens	.	.	0.3	2.5	.	.	0.1	22.0	0.5	8.0	16.3	41.1	.	.	5.1	17.2	18.9	38.7	3.9	27.7	.	.	.	.	.	.	0.4	10.9	0.0	25.8	.	.	5.1	23.1
Escherichia	.	.	4.1	5.8	.	.	2.1	3.0	.	.	26.4	29.0	0.2	0.2	0.2	1.2	0.2	0.8	0.0	3.2	.	.	40.5	42.4	.	.	11.0	18.8	.	.	0.4	0.5	2.7	3.4
Escherichia coli, ETEC	.	.	.	.	.	.	.	.	.	.	.	.	.	.	.	.	.	.	.	.	.	.	10.4	16.8	.	.	6.9	53.3	.	.	.	.	31.2	38.7
Escherichia coli, O157 STEC	.	.	.	.	.	.	6.7	9.8	.	.	33.0	41.3	0.5	0.8	0.7	4.0	0.8	2.5	0.1	10.4	.	.	18.0	22.5	.	.	19.3	31.5	.	.	1.1	1.7	.	.
Escherichia coli, non-O157 STEC	.	.	.	.	.	.	.	.	.	.	29.7	29.7	.	.	.	.	.	.	.	.	.	.	62.2	62.2	.	.	8.1	8.1	.	.	.	.	.	.
Escherichia coli, other	.	.	70.6	100	.	.	.	.	.	.	.	.	.	.	.	.	.	.	.	.	.	.	.	.	.	.	.	.	.	.	.	.	.	.
Listeria monocytogenes	.	.	.	.	.	.	15.7	16.3	.	.	1.2	35.6	.	.	4.2	38.1	38.4	72.8	.	.	.	.	.	.	.	.	.	.	.	.	6.0	6.0	.	.
Mycobacterium bovis	.	.	.	.	.	.	.	.	.	.	.	.	.	.	.	.	.	.	.	.	.	.	.	.	.	.	.	.	.	.	.	.	.	.
Salmonella eterica	0.6	3.6	0.1	0.7	0.3	0.3	6.0	18.6	11.6	29.0	3.5	14.9	0.3	2.5	3.6	11.4	10.1	29.2	.	.	.	.	6.0	11.5	0.1	1.4	1.9	18.5	0.8	14.8	1.8	2.1	9.6	22.9
Ser†. Enteritidis	0.2	9.1	0.3	2.2	.	.	0.5	16.7	35.2	61.8	0.8	11.1	.	.	0.5	7.3	10.6	20.9	.	.	.	.	2.6	8.1	.	.	0.2	25.2	.	.	1.3	1.9	1.7	19.9
Ser†. Heidelberg	.	.	.	.	.	.	.	.	6.7	48.6	0.6	19.6	.	.	.	.	7.5	34.3	.	.	.	.	0.1	29.8	.	.	.	.	.	.	.	.	.	.
Ser†. Javiana	.	.	.	.	.	.	.	.	.	.	.	.	.	.	6.9	10.3	0.5	22.1	.	.	.	.	48.1	48.5	.	.	3.1	22.2	0.3	24.9	.	.	12.7	32.9
Ser†. Newport	.	.	.	.	.	.	7.4	12.8	.	.	9.6	15.4	.	.	5.7	8.0	8.1	13.0	.	.	.	.	10.0	17.1	.	.	4.8	13.9	4.2	13.4	.	.	33.6	40.8
Ser†. Typhi	.	.	.	.	100	100	.	.	.	.	.	.	.	.	.	.	.	.	.	.	.	.	.	.	.	.	.	.	.	.	.	.	.	.
Ser†. Typhimurium	1.5	2.3	.	.	.	.	12.5	31.4	1.0	16.6	4.2	19.8	0.7	6.8	5.5	19.4	13.3	44.2	.	.	.	.	.	.	0.3	3.9	2.2	18.9	0.2	29.6	4.0	4.2	6.6	20.8
Other non-typhoidal	0.6	1.8	0.1	1.1	.	.	0.9	14.7	1.4	15.5	6.7	13.2	0.4	3.9	5.9	11.5	10.3	19.6	2.5	12.7	.	.	9.3	21.5	.	.	0.7	13.7	0.2	14.8	4.2	7.0	18.9	29.9
Shigella spp.	1.4	1.4	.	.	0.2	0.2	0.2	22.6	0.1	16.6	2.1	7.4	.	.	0.1	1.8	1.4	12.2	0.3	23.5	.	.	1.7	11.2	.	.	16.0	44.6	.	.	.	.	23.2	57.5
Staphylococcus aureus	0.2	1.9	0.5	2.6	0.3	1.7	0.9	24.9	0.3	29.9	3.9	18.9	0.0	4.9	22.9	51.5	9.4	40.6	0.1	28.3	.	.	.	.	.	.	.	.	0.6	23.9	.	.	0.0	12.9
Streptococcus spp. group A	.	.	.	.	.	.	.	.	.	.	.	.	.	.	.	.	100	100	.	.	.	.	.	.	.	.	.	.	.	.	.	.	.	.
Vibrio	3.7	7.5	13.5	18.9	70.8	73.6	.	.	.	.	.	.	.	.	.	.	2.6	2.8	.	.	.	.	.	.	.	.	.	.	.	.	.	.	.	.
V. cholerae, toxigenic	.	.	50.0	50.0	50.0	50.0	.	.	.	.	.	.	.	.	.	.	.	.	.	.	.	.	.	.	.	.	.	.	.	.	.	.	.	.
V. parahaemolyticus	.	.	20.3	28.4	67.4	71.6	.	.	.	.	.	.	.	.	.	.	3.9	4.2	.	.	.	.	.	.	.	.	.	.	.	.	.	.	.	.
V. spp., other	11.1	22.2	.	.	77.8	77.8	.	.	.	.	.	.	.	.	.	.	.	.	.	.	.	.	.	.	.	.	.	.	.	.	.	.	.	.
V. vulnificus	.	.	.	.	.	.	.	.	.	.	.	.	.	.	.	.	.	.	.	.	.	.	.	.	.	.	.	.	.	.	.	.	.	.

Etiologic Agent	Fish min	Fish max	Crust. min	Crust. max	Mollusks min	Mollusks max	Dairy min	Dairy max	Eggs min	Eggs max	Beef min	Beef max	Game min	Game max	Pork min	Pork max	Poultry min	Poultry max	Grains-Beans min	Grains-Beans max	Oils-Sugars min	Oils-Sugars max	Fruits-Nuts min	Fruits-Nuts max	Fungi min	Fungi max	Leafy min	Leafy max	Root min	Root max	Sprout min	Sprout max	Vine-Stalk min	Vine-Stalk max
Yersinia enterocolitica	.	.	.	.	.	.	0	0	.	.	.	.	.	.	86.2	100	.	.	.	.	.	.	.	.	.	.	.	.	.	.	.	.	.	.
Chemical	8.4	8.5	0.1	0.1	0.2	0.2	0.0	1.1	0.1	0.9	0.0	0.1	0.1	0.1	0.0	0.1	0.6	1.4	0.2	1.8	0.0	0.9	1.5	1.7	0.2	0.4	0.1	1.4	0.0	1.4	.	.	0.1	0.8
Marine biotoxins	95.4	97.3	0.5	0.9	2.2	2.5	.	.	.	.	.	.	.	.	.	.	.	.	.	.	.	.	.	.	.	.	.	.	.	.	.	.	.	.
Mycotoxins	.	.	.	.	.	.	.	.	.	.	.	.	.	.	.	.	.	.	.	.	.	.	58.6	58.6	35.9	41.4	.	.	.	.	.	.	.	.
Other chemicals	5.4	5.4	0.5	0.5	.	.	0.7	20.8	2.8	17.0	0.6	2.8	1.6	2.8	0.2	1.4	12.2	25.5	2.9	33.5	0.4	16.6	22.0	27.4	0.2	3.0	2.1	26.8	0.2	26.4	.	.	1.0	14.9
Parasitic	0.1	0.1	.	.	9.4	9.4	.	.	.	.	.	.	0.0	0.0	.	.	.	.	.	.	.	.	7.8	8.3	.	.	0.0	0.1	.	.	.	.	0.0	0.2
Anisakis simplex	100	100	.	.	.	.	.	.	.	.	.	.	.	.	.	.	.	.	.	.	.	.	.	.	.	.	.	.	.	.	.	.	.	.
Cryptosporidium spp.	.	.	.	.	.	.	.	.	.	.	.	.	.	.	.	.	.	.	.	.	.	.	94.7	100	.	.	.	.	.	.	.	.	.	.
Cyclospora cayetanensis	.	.	.	.	.	.	.	.	.	.	.	.	.	.	.	.	.	.	.	.	.	.	82.9	88.9	.	.	0.7	5.6	.	.	.	.	0.3	15.3
Giardia intestinalis	.	.	.	.	100	100	.	.	.	.	.	.	.	.	.	.	.	.	.	.	.	.	.	.	.	.	.	.	.	.	.	.	.	.
Toxoplasma gondii	.	.	.	.	.	.	.	.	.	.	.	.	.	.	.	.	.	.	.	.	.	.	.	.	.	.	.	.	.	.	.	.	.	.
Trichinella spp.	.	.	.	.	.	.	.	.	.	.	.	.	90.9	100	.	.	.	.	.	.	.	.	.	.	.	.	.	.	.	.	.	.	.	.
Viral	0.2	0.5	0.0	0.3	0.3	0.4	0.3	5.1	0.1	5.4	0.2	2.2	0.0	0.8	0.2	2.2	0.2	2.8	0.1	4.9	0.0	4.7	1.0	3.5	.	.	1.3	7.3	0.1	5.7	.	.	0.2	5.4
Astrovirus	.	.	.	.	.	.	.	.	.	.	.	.	.	.	.	.	.	.	.	.	.	.	.	.	.	.	.	.	.	.	.	.	.	.
Hepatitis A virus	.	.	0.5	1.0	3.1	3.1	0.6	6.0	.	.	.	.	.	.	.	.	.	.	.	.	.	.	2.9	8.2	.	.	57.7	65.6	23.3	31.5	.	.	1.8	7.9
Norovirus	1.2	3.4	0.2	2.0	1.8	2.5	2.2	36.0	0.9	37.9	1.2	15.3	0.0	5.7	1.2	15.3	1.6	19.7	0.7	34.1	0.2	33.1	7.0	24.9	.	.	8.9	51.3	0.7	39.8	.	.	1.3	38.3
Rotavirus	4.3	98.3	.	.	1.7	1.7	.	.	.	.	.	.	.	.	.	.	.	.	.	.	.	.	.	.	.	.	.	.	.	.	.	.	.	.
Sapovirus	.	.	.	.	.	.	.	.	.	.	.	.	.	.	.	.	.	.	46.2	46.2	.	.	.	.	.	.	53.8	53.8	.	.	.	.	.	.
TOTAL	2.3	4.1	0.4	1.9	2.8	3.3	7.2	31.7	1.3	26.1	3.6	15.8	0.1	3.8	3.2	14.8	5.0	20.6	1.1	24.8	0.1	19.1	6.7	18.6	0.0	0.2	6.6	34.5	0.8	29.0	0.3	0.5	3.4	29.1

†STEC=Shiga toxin-producing *Escherichia coli;* ETEC=Enterotoxigenic *Escherichia coli;* other= diarrheagenic *E. coli* other than STEC and ETEC; ser.=serotype.

Technical Appendix 1 Table 4. Number of foodborne disease outbreaks that were attributed to each food commodity, by etiologic agent, using outbreak data from 1998 through 2008

		Aquatic animals			Land animals						Plants							
			Shellfish				Meat-Poultry						Produce					
							Meat							Vegetables				
Etiologic agent	TOTAL	Fish	Crustacean	Mollusk	Dairy	Eggs	Beef	Game	Pork	Poultry	Grains-Beans	Oils-Sugars	Fruits-Nuts	Fungi	Leafy	Root	Sprout	Vine-Stalk
Bacterial	2,469	81	97	85	596	586	593	120	413	713	742	361	207	29	501	554	45	492
Bacillus cereus	197	5	8	3	35	59	27	8	21	52	131	64	17	5	54	80	3	37
Brucella spp.	4	.	.	.	4	.	.	.	.	.	.	.	.	.	.	.	.	.
Campylobacter spp.	138	2	1	4	78	11	10	3	10	37	12	8	8	1	20	14	1	17
Clostridium botulinum	30	15	2	2	.	1	3	2	1	2	3	1	1	1	4	8	.	6
Clostridium perfringens	461	6	9	4	93	44	199	22	98	173	157	55	21	6	64	111	1	102
Escherichia	206	2	3	1	28	23	109	7	12	11	43	15	22	1	67	26	8	39
E. coli, ETEC	11	1	.	.	4	5	2	.	1	2	5	3	2	.	7	5	.	4
E. coli, O157 STEC	186	1	1	1	23	17	103	6	10	8	36	11	18	1	59	20	8	34
E. coli, non-O157 STEC	6	.	.	.	.	.	3	.	.	.	.	.	2	.	1	.	.	.
E. coli, other	3	.	2	.	1	1	1	1	1	1	2	1	.	.	.	1	.	1
Listeria monoctyogenes	21	1	.	.	7	1	6	.	6	10	1	.	.	.	2	1	1	1
Salmonella	877	28	26	9	210	324	128	38	115	271	211	114	103	11	160	169	29	160
Ser†. Enteritidis	284	12	13	4	78	192	32	7	24	61	81	45	23	2	50	51	7	42
Ser†. Heidelberg	66	1	.	.	29	36	9	4	7	22	25	21	10	1	8	9	.	10

		Aquatic animals			Land animals						Plants							
			Shellfish				Meat-Poultry						Produce					
							Meat							Vegetables				
Etiologic agent	TOTAL	Fish	Crustacean	Mollusk	Dairy	Eggs	Beef	Game	Pork	Poultry	Grains-Beans	Oils-Sugars	Fruits-Nuts	Fungi	Leafy	Root	Sprout	Vine-Stalk
Ser[†]. Javiana	17	.	.	.	3	1	1	.	3	4	4	1	4	.	4	5	.	6
Ser[†]. Newport	58	.	.	.	11	4	12	3	6	15	9	3	13	.	11	10	.	15
Ser[†]. Typhimurium	106	3	2	1	29	21	15	5	15	39	28	18	14	4	22	25	3	20
Ser[†]. spp., other non-typhoidal	344	12	11	3	59	70	59	19	60	130	64	26	38	4	65	69	19	67
Ser[†]. Typhi	2	.	.	1	1	.	.	.	.	.	.	.	1	.	.	.	.	.
Shigella spp.	63	2	2	2	17	20	11	2	5	12	16	12	8	1	27	25	3	20
Staphylococcus aureus	384	14	15	10	118	97	98	37	137	141	161	87	24	3	98	117	1	107
Streptococcus spp. group A	1	.	.	.	.	.	.	.	.	1	.	.	.	.	.	.	.	.
Vibrio	80	6	31	50	5	6	1	1	1	2	7	5	.	.	6	3	.	1
V. cholerae, toxigenic	3	.	1	2	.	.	.	.	.	.	.	.	.	.	.	.	.	.
V. parahaemolyticus	68	4	30	41	5	6	1	1	1	2	6	5	.	.	5	3	.	1
V. spp., other	9	2	.	7	.	.	.	.	.	.	1	.	.	.	1	.	.	.
Yersinia enterocolitica	7	.	.	.	1	.	1	.	7	1	.	.	.	.	.	.	.	.
Chemical	632	526	8	14	32	21	7	5	5	10	32	29	22	18	31	34	3	19
Marine biotoxins	527	514	6	14	7	6	.	.	.	.	5	2	.	.	9	12	.	1
Mycotoxins	16	.	.	.	1	.	.	.	.	.	.	.	3	13	1	1	.	.
Other chemicals	89	12	2	.	24	15	7	5	5	10	27	27	19	5	21	21	3	18
Parasitic	33	2	.	1	.	4	2	8	3	4	6	6	11	1	9	8	1	7
Anisakis simplex	1	1	.	.	.	.	.	.	.	.	.	.	.	.	.	.	.	.
Cryptosporidium spp.	3	.	.	.	.	1	.	.	.	.	1	1	1	.	1	2	.	1
Cyclospora cayetanensis	16	1	.	.	.	2	1	.	1	1	4	4	9	.	5	3	.	4
Giardia intestinalis	4	.	.	1	.	1	.	.	.	2	1	1	.	1	3	3	1	2
Trichinella spp.	9	.	.	.	.	.	1	8	2	1	.	.	1	.	.	.	.	.
Viral	1,455	49	43	69	552	518	250	104	221	326	527	491	286	83	741	540	69	536
Hepatitis A virus	29	1	2	1	7	6	2	.	1	2	2	6	8	5	16	11	5	9
Norovirus	1419	46	41	67	545	510	247	104	219	321	522	483	278	78	722	528	64	525
Rotavirus	5	2	.	1	.	2	1	.	1	3	2	2	.	.	2	1	.	2
Sapovirus	2	.	.	.	.	.	.	.	.	.	1	.	.	.	1	.	.	.
Total	4,589	658	148	169	1,180	1,129	852	237	642	1,053	1,309	889	477	131	1,286	1,138	118	1,057

[†]STEC=Shiga toxin-producing *Escherichia coli;* ETEC=Enterotoxigenic *Escherichia coli;* other= diarrheagenic *E. coli* other than STEC and ETEC; ser.=serotype.

Technical Appendix 1 Table 5. Comparison of rank order of illnesses, hospitalizations, and deaths attributed to food commodities when adjusting the attribution algorithm to account for variation among the number of outbreak illnesses.

A) Comparison of rank order and percentage of **illnesses** attributed to food commodities when adjusting the attribution algorithm to account for variation among the number of outbreak illnesses.

	Attributed Illnesses As a Function of Outbreak-associated Illnesses Included in Model									
	All Outbreaks, all illnesses[*]		Small Outbreaks, 2–19 Illnesses per outbreak[†]		Medium Outbreaks, 10–100 Illnesses per outbreak[‡]		Large Outbreaks, ≥20 illnesses per outbreak[§]		All Outbreaks, 1 illness per outbreak[¶#]	
Commodity	Rank	%	Rank	%	Rank	%	Rank	%	Rank	%
Leafy	1	22.3	1	20.5	1	23.4	1	22.7	1	22.3
Dairy	2	13.8	3	11.5	4	9.4	2	14.2	3	11.2
Fruits-Nuts	3	11.7	6	7.0	2	12.3	3	11.9	4	8.6
Poultry	4	9.8	2	14.4	3	11.1	4	9.1	2	13.3
Vine	5	7.9	9	5.3	8	6.2	5	8.1	10	4.9
Beef	6	6.6	4	8.3	5	7.2	7	6.0	5	8.5
Eggs	7	6.0	8	5.5	6	6.9	6	6.1	8	5.3
Pork	8	5.4	7	6.6	7	6.7	8	5.5	7	6.3
Grains-Beans	9	4.5	5	7.9	9	4.7	11	3.6	6	6.6
Root	10	3.6	11	3.2	10	4.5	10	3.6	11	3.4
Mollusk	11	3.0	10	3.5	13	1.3	13	1.9	9	4.0
Fish	12	2.7	12	2.3	12	1.9	12	2.5	12	2.5
Undetermined	13	1.1	13	1.8	11	2.4	9	3.7	13	1.1
Oils-Sugars	14	0.7	14	0.8	14	0.9	14	0.6	15	0.7
Crustacean	15	0.5	15	0.7	15	0.6	16	0.4	14	0.7
Sprout	16	0.3	17	0.2	16	0.5	15	0.4	16	0.3
Game	17	0.1	16	0.5	17	0.1	17	0.0	17	0.3
Fungi	18	0.1	18	0.2	18	0.0	18	0.0	18	0.1

*4,589 Outbreaks; 120,321 illnesses
†3,126 Outbreaks; 21,701 illnesses
‡2,244 Outbreaks; 80,368 illnesses
§1,463 Outbreaks; 98,620 illnesses
¶4,589 Outbreaks
#Each outbreak adjusted to count only one illness per outbreak, which is equivalent to modeling attribution based on outbreak counts. Further discussion of attribution models based on outbreak counts versus outbreak illnesses can be found in Technical Appendix 2 (wwwnc.cdc.gov/EID/article/19/3/11-1866-Techapp2.pdf).

B) Comparison of rank order of **hospitalizations** attributed to food commodities when adjusting the attribution algorithm to account for variation among the number of outbreak illnesses.

	Attributed Hospitalizations As a Function of Outbreak Illnesses Included in Model									
	All Outbreaks, all illnesses*		Small Outbreaks, 2–19 Illnesses per outbreak†		Medium Outbreaks, 10–100 Illnesses per outbreak‡		Large Outbreaks, ≥20 illnesses per outbreak§		All Outbreaks, 1 illness per outbreak¶#	
Commodity	Rank	%	Rank	%	Rank	%	Rank	%	Rank	%
Dairy	1	16.2	2	15.3	3	13.0	1	16.1	2	14.6
Leafy	2	13.5	3	13.0	1	14.2	2	13.7	3	13.4
Poultry	3	11.5	1	17.8	2	13.5	3	11.4	1	17.0
Vine	4	10.5	8	5.3	5	7.5	5	11.2	7	4.8
Fruits-Nuts	5	10.1	9	5.3	9	8.6	4	10.3	9	6.4
Undetermined	6	8.1	5	8.5	4	9.7	6	10.2	5	8.1
Eggs	7	7.1	4	8.5	6	7.9	7	7.0	4	8.3
Beef	8	5.4	6	6.6	7	6.9	9	4.8	6	7.1
Pork	9	5.1	7	6.1	8	6.9	8	5.0	8	6.2
Fish	10	2.9	11	2.7	12	2.0	11	2.2	11	3.0
Root	11	2.6	12	2.5	10	4.0	10	2.5	12	2.7
Grains-Beans	12	2.5	10	3.4	11	2.6	12	2.1	10	3.0
Mollusk	13	2.5	13	2.3	14	0.6	13	1.7	13	2.8
Sprout	14	1.2	15	0.7	13	1.7	14	1.4	14	1.2
Oils-Sugars	15	0.3	17	0.4	15	0.4	15	0.3	17	0.4
Crustacean	16	0.2	14	0.5	16	0.2	17	0.1	15	0.5
Game	17	0.2	16	0.9	17	0.2	16	0.1	16	0.6
Fungi	18	0.1	18	0.3	18	0.0	18	0.0	18	0.2

*4,589 Outbreaks; 120,321 illnesses
†3,126 Outbreaks; 21,701 illnesses
‡2,244 Outbreaks; 80,368 illnesses
§1,463 Outbreaks; 98,620 illnesses
¶4,589 Outbreaks
#Each outbreak adjusted to count only one illness per outbreak, which is equivalent to modeling attribution based on outbreak counts. Further discussion of attribution models based on outbreak counts versus outbreak illnesses can be found in Technical Appendix 2 (wwwnc.cdc.gov/EID/article/19/3/11-1866-Techapp2.pdf).

C) Comparison of rank order of **deaths** attributed to food commodities when adjusting the attribution algorithm to account for variation among the number of outbreak illnesses.

	Attributed Deaths As a Function of Outbreak Illnesses Included in Model									
	All Outbreaks, all illnesses*		Small Outbreaks, 2–19 Illnesses per outbreak†		Medium Outbreaks, 10–100 Illnesses per outbreak‡		Large Outbreaks, >20 illnesses per outbreak§		All Outbreaks, 1 illness per outbreak¶#	
Commodity	Rank	%	Rank	%	Rank	%	Rank	%	Rank	%
Undetermined	1	25.2	1	25.5	1	26.6	1	27	1	25.2
Poultry	2	19.1	3	14.6	2	21.3	2	24	2	17.2
Dairy	3	9.7	2	15.9	3	8.0	5	7	3	11.9
Vine	4	7.0	10	3.2	10	4.6	4	7	10	3.0
Fruits-Nuts	5	6.4	9	3.0	7	5.2	3	7	9	3.9
Leafy	6	6.0	5	6.3	4	6.6	6	6	5	6.1
Pork	7	5.7	4	8.4	5	6.7	9	4	4	7.8
Fish	8	4.9	7	5.0	9	4.4	8	4	8	5.2
Eggs	9	4.9	6	6.1	6	5.5	7	5	6	6.0
Beef	10	3.8	8	4.9	8	4.5	10	3	7	5.6
Sprout	11	1.9	15	0.5	11	2.4	11	2	13	1.7
Grains-Beans	12	1.9	11	2.0	12	1.8	12	1	11	1.9
Root	13	1.4	12	1.6	13	1.7	13	1	12	1.7
Mollusk	14	1.4	13	1.3	14	0.5	14	1	14	1.4
Game	15	0.2	14	0.7	15	0.2	17	0	16	0.4
Oils-Sugars	16	0.2	18	0.3	17	0.2	15	0	17	0.4
Crustacean	17	0.2	16	0.4	16	0.2	16	0	15	0.4
Fungi	18	0.1	17	0.4	18	0.0	18	0	18	0.3

[1] Scallan E, Hoekstra RM, Angulo FJ, Tauxe RV, Widdowson MA, Roy SL, et al. Foodborne illness acquired in the United States--major pathogens. Emerg Infect Dis. 2011 Jan;17(1):7-15.
*4,589 Outbreaks; 120,321 illnesses
†3,126 Outbreaks; 21,701 illnesses
‡2,244 Outbreaks; 80,368 illnesses
§1,463 Outbreaks; 98,620 illnesses
¶4,589 Outbreaks
#Each outbreak adjusted to count only one illness per outbreak, which is equivalent to modeling attribution based on outbreak counts. Further discussion of attribution models based on outbreak counts versus outbreak illnesses can be found in Technical Appendix 2 (wwwnc.cdc.gov/EID/article/19/3/11-1866-Techapp2.pdf).
