# Supplementary material for: Attribution of Foodborne Illnesses, Hospitalizations, and Deaths to Food Commodities by using Outbreak Data, United States, 1998–2008
Source: Emerg Infect Dis. 2013 Mar;19(3):407–15. doi: 10.3201/eid1903.111866 (PMC3647642; doi:10.3201/eid1903.111866)
Supplement: Technical Appendix 2 — Additional methods related to attributing human illnesses to food commodities. [file 11-1866-Techapp-s2.pdf]

Article DOI: http://dx.doi.org/10.3201/eid1903.111866

Attribution of Foodborne Illnesses, Hospitalizations, and Deaths to Food Commodities Using Outbreak Data, United States, 1998–2008

Technical Appendix 2

In this appendix, we reframe the problem of attributing human illness to food commodities in a more mathematical fashion to provide additional documentation and insight into our method.

The population of interest is defined as the set of all instances of symptomatic human illness caused by consuming contaminated food in the United States during some time period. We observe a set of reports describing outbreaks linked to particular etiologic agents and food exposures. The outbreaks analyzed are restricted to those for which there is a single etiologic agent and the ingredients of the contaminated food(s) can be characterized. We then map the information in those reports to create a set of outbreak-specific multivariate observations containing the specific etiologic agent, the contaminated food(s), and the number of illnesses for each outbreak. Next, each food is mapped to a set of ingredients among a specific 17-category food commodity classification scheme (1). At this point, each outbreak is identified with an etiologic agent, a number of human illnesses, and a 17-vector of 0/1 indicator variables recording whether or not the implicated food(s) included ingredients from a given commodity.

If the implicated foods contain ingredients from only one commodity, or if the contaminated ingredient is known, then that outbreak is classified as simple, whereas if the ingredients are from multiple commodities, and the contaminated ingredient is not known, then that outbreak is classified as complex.

Estimated total number of illnesses ($\widehat{\boldsymbol{T}}$) attributed to each of the 17 commodities by using the three estimators described in the methods section of the manuscript, minimum, most probable (MP) and maximum, are given by the following equations:

$$
\begin{aligned}
\widehat{\boldsymbol{T}} &= \left(\widehat{\boldsymbol{T}}_{\mathbf{1}}, \widehat{\boldsymbol{T}}_{\mathbf{2}}, \cdots, \widehat{\boldsymbol{T}}_{\boldsymbol{j}}, \cdots, \widehat{\boldsymbol{T}}_{\mathbf{17}}\right)' \\
\widehat{\boldsymbol{T}}(\boldsymbol{minimum}) &= \sum_{i=\mathbf{1}}^{K} b_i \times \left[\frac{\boldsymbol{n}_i'\boldsymbol{S}_i}{\left(\boldsymbol{n}_i'\boldsymbol{S}_i + \boldsymbol{m}_i'[diag(\boldsymbol{C}_i\boldsymbol{q}_i)]^{-1}\boldsymbol{C}_i diag(\boldsymbol{q}_i)\right)\mathbf{1}_{17}}\right]' \\
&= \boldsymbol{where}\ \boldsymbol{q}_i = \frac{\boldsymbol{n}_i'\boldsymbol{S}_i}{(\boldsymbol{n}_i'\boldsymbol{S}_i)\mathbf{1}_{17}} \\
\widehat{\boldsymbol{T}}(\boldsymbol{MP}) &= \sum_{i=\mathbf{1}}^{K} b_i \times \left[\frac{\boldsymbol{n}_i'\boldsymbol{S}_i + \boldsymbol{m}_i'[diag(\boldsymbol{C}_i\boldsymbol{q}_i)]^{-1}\boldsymbol{C}_i diag(\boldsymbol{q}_i)}{\left(\boldsymbol{n}_i'\boldsymbol{S}_i + \boldsymbol{m}_i'[diag(\boldsymbol{C}_i\boldsymbol{q}_i)]^{-1}\boldsymbol{C}_i diag(\boldsymbol{q}_i)\right)\mathbf{1}_{17}}\right]' \\
\widehat{\boldsymbol{T}}(\boldsymbol{maximum}) &= \sum_{i=\mathbf{1}}^{K} b_i \times \left[\frac{\boldsymbol{n}_i'\boldsymbol{S}_i + \boldsymbol{m}_i'\boldsymbol{C}_i}{\left(\boldsymbol{n}_i'\boldsymbol{S}_i + \boldsymbol{m}_i'\left[diag\left(\boldsymbol{q}\boldsymbol{C}_{i_i}\right)\right]^{-1}\boldsymbol{C}_i diag(\boldsymbol{q}_i)\right)\mathbf{1}_{17}}\right]'
\end{aligned}
$$

where i indexes the etiologic agents, b_i are the public health burdens (e.g., numbers of illnesses, hospitalizations, or deaths), $\boldsymbol{n}_i$ and $\boldsymbol{m}_i$ are column vectors with elements equal to the numbers of reported cases in simple and complex outbreaks respectively, $\boldsymbol{S}_i$ and $\boldsymbol{C}_i$ are 0/1 matrices corresponding to simple and complex outbreaks of etiology $\boldsymbol{i}$ with rows indexing outbreaks and columns corresponding to whether or not a given commodity was represented by ingredients of the contaminated food or foods from the outbreak. In some instances, one or both of $\boldsymbol{S}_i$ and $\boldsymbol{C}_i$ will be null matrices because no outbreaks of a given etiology were reported in that category. In addition, when $\boldsymbol{S}_i$ is null, $\boldsymbol{C}_i$ is defined to be null and the term in the summation is defined to be 0. When a column of $\boldsymbol{S}_i$ is null then the corresponding column of $\boldsymbol{C}_i$ is null. This follows from the general rule that no illnesses from complex food outbreaks due to a given etiologic agent will be allocated to commodities that are not represented among the simple food outbreaks due to that agent. The vector $\mathbf{1}_{\mathbf{17}}$ is a column vector of 1's with dimension 17.

Estimated proportions ($\hat{\boldsymbol{p}}$) of all illnesses attributed to the 17 defined commodity groups are given by related equations:

$$\hat{\boldsymbol{p}} = \left(\hat{\boldsymbol{p}}_1, \hat{\boldsymbol{p}}_2, \cdots, \hat{\boldsymbol{p}}_j, \cdots, \hat{\boldsymbol{p}}_{17}\right)'$$

$$\hat{\boldsymbol{p}}(\boldsymbol{minimum}) = \sum_{i=1}^{K} \frac{b_i}{\sum b_i} \times \left[\frac{\boldsymbol{n}_i'\boldsymbol{S}_i}{\left(\boldsymbol{n}_i'\boldsymbol{S}_i + \boldsymbol{m}_i'[diag(\boldsymbol{C}_i\boldsymbol{q}_i)]^{-1}\boldsymbol{C}_i diag(\boldsymbol{q}_i)\right)\mathbf{1}_{17}}\right]'$$

$$\boldsymbol{where}\ \boldsymbol{q}_i = \frac{\boldsymbol{n}_i'\boldsymbol{S}_i}{(\boldsymbol{n}_i'\boldsymbol{S}_i)\mathbf{1}_{17}}$$

$$\hat{\boldsymbol{p}}(\boldsymbol{MP}) = \sum_{i=1}^{K} \frac{b_i}{\sum b_i} \times \left[\frac{\boldsymbol{n}_i'\boldsymbol{S}_i + \boldsymbol{m}_i'[diag(\boldsymbol{C}_i\boldsymbol{q}_i)]^{-1}\boldsymbol{C}_i diag(\boldsymbol{q}_i)}{\left(\boldsymbol{n}_i'\boldsymbol{S}_i + \boldsymbol{m}_i'[diag(\boldsymbol{C}_i\boldsymbol{q}_i)]^{-1}\boldsymbol{C}_i diag(\boldsymbol{q}_i)\right)\mathbf{1}_{17}}\right]'$$

$$\hat{\boldsymbol{p}}(\boldsymbol{maximum}) = \sum_{i=1}^{K} \frac{b_i}{\sum b_i} \times \left[\frac{\boldsymbol{n}_i'\boldsymbol{S}_i + \boldsymbol{m}_i'\boldsymbol{C}_i}{\left(\boldsymbol{n}_i'\boldsymbol{S}_i + \boldsymbol{m}_i'[diag(\boldsymbol{C}_i\boldsymbol{q}_i)]^{-1}\boldsymbol{C}_i diag(\boldsymbol{q}_i)\right)\mathbf{1}_{17}}\right]'$$

Note that the minimum estimated totals sum to less than the total estimated number of illnesses, $\sum b_i$, and the maximum estimated totals sum to more than $\sum b_i$, when there are attributable illnesses due to complex foods. The same is true of the minimum estimated proportions and maximum estimated proportions; they sum to less than and more than 1, respectively. Thus the minimum and maximum estimates cannot be interpreted as estimators of the set of commodity attribution fractions by themselves. Only the most probable estimates perform that function. With these limitations in mind, there are several things to note about the equations. The minimum, MP, and maximum estimates reflect the different allocations of observed illnesses from complex outbreaks that are added to illnesses observed from simple outbreaks. The minimum estimates, $\hat{\boldsymbol{T}}(\boldsymbol{minimum})$, evaluate the minimal level of individual commodity attribution, before adding the contribution from complex outbreak data. They do not represent what might be called simple outbreak attribution, because while the numerators are based on allocating simple food outbreak illnesses, the denominators reflect both simple and complex illnesses. The MP estimates, $\hat{\boldsymbol{T}}(\boldsymbol{MP})$, add in the information from complex outbreaks in a weighted fashion, and the maximum estimates, $\hat{\boldsymbol{T}}(\boldsymbol{maximum})$, evaluate the maximal level of individual commodity attribution derived from the addition of all complex food illnesses allocated to single commodities.

The MP equation in its proportional formulation, $\hat{\boldsymbol{p}}(\boldsymbol{MP})$, allows comparison of commodity attributions between models with different burdens. Also, by varying the set of

etiologies being summed from the complete set to subsets, comparisons of commodity attributions can be made between, for example, bacterial etiologies and viral etiologies. As mentioned, the minimum and maximum equations are set up to provide bounds on attributions to individual commodities. The vectors of proportions, $\widehat{\boldsymbol{p}}(\boldsymbol{minimum})$ and$\widehat{\boldsymbol{p}}(\boldsymbol{maximum})$, do not provide comparisons of attributions across the 17 commodities. Note that the vector, $\boldsymbol{q_i}$, is composed of a set of proportions that sum to 1, and does correspond to the attribution of illnesses from only simple food outbreaks due to a specific etiologic agent.

The dimensions of the vectors and matrices in the equations vary with etiologic agent; for each etiology and each set of simple and complex outbreaks, the number of rows is defined by the number of reported outbreaks satisfying the requirements for estimation. This embeds a hidden level of uncertainty in the output of the equations in that for some etiologic agents the number of reported outbreaks is small. This is a different type of uncertainty from that captured by the range of values described by the three estimators. This model does not incorporate uncertainties associated with the accuracy of reported etiologies, food exposures, or numbers of ill persons.

Mathematically, the equations are quite general. For example, the MP equation in its proportional form can be looked at as

$$\begin{aligned}\widehat{\boldsymbol{p}}(\boldsymbol{MP}) \quad &= \quad \sum_{\boldsymbol{i=1}}^{K} \frac{b_i}{\sum b_i} \times \left[\frac{\boldsymbol{n}_i'\boldsymbol{S}_i + \boldsymbol{m}_i'[diag(\boldsymbol{C}_i\boldsymbol{q}_i)]^{-1}\boldsymbol{C}_i diag(\boldsymbol{q}_i)}{\left(\boldsymbol{n}_i'\boldsymbol{S}_i + \boldsymbol{m}_i'[diag(\boldsymbol{C}_i\boldsymbol{q}_i)]^{-1}\boldsymbol{C}_i diag(\boldsymbol{q}_i)\right)\boldsymbol{1}_{17}} \right]' \\ &= \quad \sum_{etiologies} \boldsymbol{P}(etiology) \times \boldsymbol{P}(comm1, comm2, \dots, comm17|etiology)\end{aligned}$$

where $\boldsymbol{P}(etiology)$ are weights in proportional form given to each etiology and$\boldsymbol{P}(comm1, comm2, \dots, comm17|etiology)$ are commodity proportions derived from reported numbers of illnesses for each etiology. We have made specific choices for these quantities; other possible choices have been reported in the literature. For example costs have been used to weight the etiologies(2) and outbreak counts have been used as the basis for computing the commodity proportions.(3) It is useful to note that using outbreak counts as the basis for computing commodity proportions is equivalent to using reported numbers of illnesses

if all outbreaks are the same size. For example, the two calculations applied to poultry compare as follows

$$Poultry\ attribution\ proportion = \frac{\text{sum of reported illnesses from poultry outbreaks}}{\text{sum of all reported illnesses}}$$

which can also be written as

$$Poultry\ attribution\ proportion = \frac{\text{number of poultry outbreaks} \times \text{average of reported illnesses from poultry outbreaks}}{\text{total number of outbreaks} \times \text{average of reported illnesses from all outbreaks}}$$

versus

$$Poultry\ attribution\ proportion = \frac{\text{number of poultry outbreaks}}{\text{total number of outbreaks}}$$

When all outbreaks are the same size, the averages of the numerator and denominator of the middle equation equal that constant size, and they cancel, making illness-based and outbreak-based proportions the same. The equations show that the equivalence holds more generally; if the average outbreak size for a commodity equals the overall average, then the numerator and denominator averages cancel. If each commodity average outbreak size equals the overall average, then all commodity attribution proportions will be the same, and illness-based and outbreak-based attributions are the same. Although this discussion focuses on source attribution for simple foods, the same concepts apply to source attribution for complex foods. Differences between outbreak-associated illness-based and outbreak-based attribution proportions reflect differences in outbreak sizes among commodities. The degree to which one or the other measure, outbreak-associated illnesses or outbreaks, reflect illnesses as they are caused by commodities in overall domestic foodborne illness is a subject for future research.

Expanded Methods

In this appendix, as a complement to the technical appendix, we provide a narrative description and examples of key elements of our method. To estimate illnesses attributable to specific commodities from reports of outbreaks of foodborne illnesses, we 1) attribute illnesses to specific commodities for each etiologic agent, and 2) sum the etiology-specific estimates, weighted by estimates of the number of illnesses (i.e., illnesses, hospitalizations, or deaths) for each etiology.

Attributing illnesses to specific commodities for each etiologic agent

To determine etiologic agent-specific attribution, we wanted to sum the number of illnesses attributed to specific commodities implicated in foodborne disease outbreaks caused by each agent. For outbreaks in which the implicated food(s) contained ingredients from a single commodity, this can be done easily because each outbreak contributes illnesses to a single commodity. For outbreaks in which the implicated food(s) contained ingredients from more than one commodity this cannot be done until illnesses are allocated to the multiple commodities in some fashion. To use data from commodities in both simple and complex foods, we calculated an estimate that sums the number of illnesses from simple food outbreaks attributed to specific commodities and adds to those sums partitioned numbers of illnesses from complex food outbreaks. The partitioning is based on proportions observed in simple food outbreaks. We refer to this estimate as the most probable estimate (MP).

To indicate a range of possible values for the number of illnesses caused by a commodity we calculated two additional estimates based on alternate partitions of the illnesses from complex food outbreaks. The minimum estimate derives from not allocating any complex food outbreak illnesses to any commodity. The maximum estimate derives from allocating all illnesses from a complex food outbreak to each commodity associated with the outbreak, as long as that commodity was also implicated in a simple food outbreak caused by that agent (which establishes the commodity as a possible causal exposure). This last allocation counts illnesses multiple times, but that is consistent with the maximum estimate as providing an upper bound for the number of illnesses attributed to individual commodities.

In Table A, we illustrate the attribution of illnesses in a dataset of four hypothetical outbreaks of illnesses caused by Etiologic Agent X. Illnesses in simple food

outbreaks in which ground beef, lettuce, and apple juice were implicated were attributed to the commodities beef, leafy vegetables, and fruits-nuts, respectively, for all three estimates—minimum, MP, and maximum. Outbreak D was due to a complex food so no illnesses from this outbreak were included in the minimum estimate. For the maximum estimate, all six illnesses were attributed to the beef commodity (because the vehicle contained ground beef) and all six were also attributed to the leafy vegetables commodity (because the vehicle contained lettuce), but no illnesses were attributed to the vine-stalk vegetables commodity (although the vehicle contained tomato) or the grain-beans commodity (the vehicle contained bread). This is because the dataset contained at least one simple Etiologic Agent X outbreak attributed to the commodity beef, and at least one simple Etiologic Agent X outbreak attributed to the commodity leafy vegetables, but no simple Etiologic Agent X outbreak was attributed to either vine-stalk vegetables or grains-beans.

In this example, to partition the illnesses in Outbreak D into the most probable number of illnesses for each commodity, we determined the proportion of illnesses in simple food outbreaks caused by that agent that were attributed to any commodity included in the hamburger sandwich: of the illnesses in the simple food outbreaks due to these commodities, 69% were attributed to beef and 31% to leafy vegetables. We applied these proportions to the six illnesses in Outbreak D, which yielded 4 illnesses attributed to beef and 2 attributed to leafy vegetables. The crude percentage of Etiologic Agent X illnesses attributed to each commodity was calculated by summing the number of attributed illnesses and dividing by the total number of actual illnesses in all Etiologic Agent X outbreaks. Note that although the actual number of illnesses was 46, only 40 illnesses were attributed to commodities for the minimum estimate and 52 were attributed for the maximum estimate; only the MP estimate counted each illness once and only once.

Summing the etiology-specific estimates, weighted by estimated number of domestically-acquired foodborne illnesses for each etiology

To calculate the total number of illnesses attributed to each commodity, we summed the etiologic agent-specific estimates obtained by applying the proportion of illnesses for each commodity to the estimated number of domestically acquired foodborne illnesses. In Tables B1-B3, we illustrate the calculations for the number of illnesses and deaths in a dataset of two hypothetical etiologies (X and Y). In Table B1, the minimum, MP, and maximum estimates of

illnesses attributed to each commodity were calculated as above, and shown as a percentage of the total for each etiology. In Table B2, a hypothetical estimated number of US illnesses each year, by etiology, is shown in the second column. (In the manuscript these estimates are from Scallan et al.) The far-right column shows the total numbers of illnesses allocated for the minimum, MP, and maximum attribution estimates. The number of illnesses attributed to each commodity is calculated as the product of the percentage of illnesses attributed to each commodity and the estimated actual number of illnesses for each etiology. To calculate the total number of illnesses attributed to each commodity for Etiologies X and Y, we summed the number of illnesses attributed to each commodity. A similar table is shown to illustrate the method for calculating the number of deaths (Table B3). In both tables the commodity percentages are reported for the most probable estimates. Note that for a specific etiology, the percentage attributed to each commodity was the same for illnesses, hospitalizations, and deaths. However, the percentage of total illnesses and deaths attributed to each commodity is different for the summed etiologies because the estimated number of illnesses, hospitalizations, and deaths is different for each etiology. Calculations for the estimated number of hospitalizations are identical in form and are not shown.

Technical Appendix 2 Table A. Attribution of illnesses to commodities in a hypothetical dataset of Etiology X outbreaks to illustrate the method for calculating minimum, most probable (MP), and maximum estimates

Ob*	Illnesses	Implicated food	Type of food†	Estimate	No. Illnesses Attributed to Commodity: Commodity: Beef	Grains-beans	Fruits-nuts	Leafy vegetables	Total
A	22	Ground beef	Simple	**Minimum**	**22**	**0**	**0**	**0**	**22**
				**MP**	**22**	**0**	**0**	**0**	**22**
				**Maximum**	**22**	**0**	**0**	**0**	**22**
B	10	Lettuce	Simple	**Minimum**	**0**	**0**	**0**	**10**	**10**
				**MP**	**0**	**0**	**0**	**10**	**10**
				**Maximum**	**0**	**0**	**0**	**10**	**10**
C	8	Apple juice	Simple	**Minimum**	**0**	**0**	**8**	**0**	**8**
				**MP**	**0**	**0**	**8**	**0**	**8**
				**Maximum**	**0**	**0**	**8**	**0**	**8**
D	6	Hamburger sandwich‡	Complex	**Minimum**	**0**	**0**	**0**	**0**	**0**
				**MP****	**4**	**0**	**0**	**2**	**6**
				**Maximum**	**6**	**0**	**0**	**6**	**12**
Total	46			**Minimum (% of 46)**	**22 (48%)**	**0**	**8 (17%)**	**10 (22%)**	**40 (87%)**
				**MP (% of 46)**	**26 (57%)**	**0**	**8 (17%)**	**12 (26%)**	**46 (100%)**
				**Maximum (% of 46)**	**28 (61%)**	**0**	**8 (17%)**	**16 (35%)**	**52 (113%)**

*Ob, Outbreak; Minimum, minimum estimate of the number of illnesses attributed to a commodity; MP, most probable estimate of the number of illnesses attributed to a commodity; Maximum, maximum estimate of the number of illnesses attributed to a commodity.
†Illnesses in outbreaks in which the implicated food was simple were included in the minimum, maximum, and most probable estimates; illnesses in outbreaks in which the implicated foods were complex were included only in the most probable and maximum estimates.
‡Hamburger sandwich ingredients: ground beef, lettuce, tomato, bread.
** For Outbreak D, the MP estimate of illnesses due to each commodity relies on information from the simple food outbreaks due to those commodities in the dataset. The total number of outbreak-associated illnesses caused by Etiology X and due to simple foods contained in the hamburger sandwich was 32, with 22 (69%) due to beef and 10 (31%) due to leafy vegetables. Rounding to the nearest integer, the MP estimate of illnesses in outbreak D attributed to beef was 4 (69% of 6) and the MP estimate of illnesses attributed to leafy vegetables was 2 (31% of 6).

Technical Appendix 2 Table B1. Percentages of illnesses attributed to specific commodities, by etiology

Etiology	Estimate	Percentage Attributed to Commodity				
		Commodity				
		Beef	Grains-beans	Fruits-nuts	Leafy vegetable	Total
X	Minimum	48%	0%	17%	22%	87%
	MP	57%	0%	17%	26%	100%
	Maximum	61%	0%	17%	35%	113%
Y	Minimum	14%	17%	18%	23%	72%
	MP	19%	24%	25%	32%	100%
	Maximum	41%	45%	46%	51%	183%

Technical Appendix 2 Table B2. Number of illnesses attributed to specific commodities by etiology and for total

Etiology	Hypothetical actual number of illnesses	Estimate	No. Illnesses Attributed to Commodity				
			Commodity				
			Beef	Grains-beans	Fruits-nuts	Leafy vegetable	Total
X	300,000	Minimum	143,000	0	52,000	65,000	261,000
		MP	170,000	0	52,000	77,000	300,000
		Maximum	183,000	0	52,000	104,000	339,000
Y	1,000,000	Minimum	138,000	172,000	184,000	230,000	724,000
		MP	190,000	238,000	254,000	317,000	1,000,000
		Maximum	414,000	448,000	460,000	506,000	1,828,000
Total X and Y	1,300,000	Minimum	281,000	172,000	236,000	295,000	985,000
		MP (% of total)	361,000 (28%)	238,000 (18%)	306,000 (24%)	395,000 (30%)	1,300,000 (100%)
		Maximum	596,000	448,000	512,000	610,000	2,167,000

Technical Appendix 2 Table B3. Number of deaths attributed to specific commodities by etiology and for total

Etiology	Hypothetical actual number of deaths	Estimate	No. Deaths Attributed to Commodities				
			Commodity				Total
			Beef	Grains-beans	Fruits-nuts	Leafy vegetable	
X	200	Minimum	96	0	35	43	174
		MP	114	0	35	52	200
		Maximum	122	0	35	70	226
Y	50	Minimum	7	9	9	11	36
		MP	10	12	13	16	50
		Maximum	21	22	23	25	91
Total X and Y	250	Minimum	103	9	44	55	210
		MP (% of total)	123 (49%)	12 (5%)	47 (19%)	68 (27%)	250 (100%)
		Maximum	142	22	58	95	317

References

1. Painter JA, Ayers T, Woodruff R, Blanton E, Perez N, Hoekstra RM, et al. Recipes for foodborne outbreaks: a scheme for categorizing and grouping implicated foods. Foodborne Pathog Dis. 2009 Dec;6(10):1259-64.

2. Batz MB, Hoffmann S, Morris JG. Ranking the Disease Burden of 14 Pathogens in Food Sources in the United States Using Attribution Data from Outbreak Investigations and Expert Elicitation. J Food Protect. 2012 Jul;75(7):1278-91.

3. Pires SM, Vieira AR, Perez E, Lo Fo Wong D, Hald T. Attributing human foodborne illness to food sources and water in Latin America and the Caribbean using data from outbreak investigations. Int J Food Microbiol. 2012 Jan 16;152(3):129-38.
